# A Case Study on the Possibility of Extending the Service Life of the Demining Machine Belt

**DOI:** 10.3390/ma17215206

**Published:** 2024-10-25

**Authors:** Miroslav Blatnický, Ján Dižo, Marek Brůna, Marek Matejka

**Affiliations:** 1 Department of Transport and Handling Machines, Faculty of Mechanical Engineering, University of Žilina, Univerzitná 8215/1, 010 26 Žilina, Slovakia; miroslav.blatnicky@fstroj.uniza.sk (M.B.); jan.dizo@fstroj.uniza.sk (J.D.); 2 Department of Technological Engineering, Faculty of Mechanical Engineering, University of Žilina, Univerzitná 8215/1, 010 26 Žilina, Slovakia; marek.bruna@fstroj.uniza.sk

**Keywords:** applied research, fatigue life, welding, structural design

## Abstract

The operational practice of the design of the Bozena 5 demining machine has shown that its belts are the critical component that fundamentally affects the functionality of the entire machine. This article is a practical continuation and extension of the previous research results from the point of view of materials (research of the uniaxial fatigue life in bending and torsion), calculation (creation of the necessary mathematical, analytical and numerical models for the research) and construction (i.e., patented design of the belt tensioning of this machine). All these actions are aimed at a single objective—to achieve a condition that guarantees a sufficient service life without malfunctions, since repairing these machines in the field is often impossible. Therefore, this study examined the fatigue life of welded joints (uniaxial bending and torsion) of S960 QL and S500MC steels welded by MAG technology. Subsequently, the data were compared with previous results (electron and laser welds) and the influence of each type of weld on the fatigue life relative to the base material was discussed. It was found that conventional MAG technology had a more significant negative impact on the fatigue life of the base material than non-conventional technologies. This trend was particularly true for the bending stress. At the same time, the bending stress was identified by the FEM analysis as the dominant load on the belt. The maximum stress in the belt link under the considered boundary conditions was approximately 240 MPa (in bending). This stress corresponded to the continuous fatigue life (more than 10^7^ cycles) for both base materials tested (S960QL, S500MC). In the whole studied spectrum of controlled deformation amplitudes (Manson–Coffin), the life of MAG welds was lower in comparison with the base material and with welds made by unconventional technologies. All the activities carried out so far (research on microstructure, hardness, strength, residual stresses, tribological properties and fatigue life) have shown that the original belt design (S500MC) using MAG technology has significant deficiencies in the state of optimal life. It is expected that the proposed material change (use of S960QL instead of S500MC) and work with advanced technologies will bring this state significantly closer.

## 1. Introduction

Looking at the calendar, it might seem like we live in the 21st century. Unfortunately, at present, the land surface that is affected by military conflicts is increasing. All weapons used in war are evil, but there is one type of weapon more insidious than others—land mines [1,2]. Their insidiousness is that they threaten the lives of not only the direct participants in the conflict, but also civilians. Worst of all, this fact continues long after the end of the war [3,4]. Demining machines are used for the safe removal of this kind of threat [5,6,7]. The effort of this paper is to present a real product (Božena 5) of one of the leading manufacturers of demining machines—WAY Industries (WAY Industries, Inc., Krupina, Slovakia).

**Figure 1:**
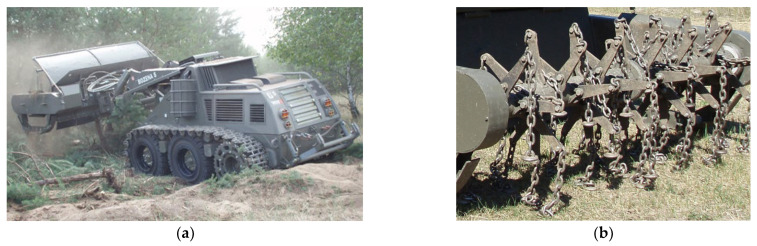
(**a**) Demining machine Božena 5; (**b**) Detail of the rotating cylinder with chains and flails.

The series of demining machines, starting with the designation Božena 1, began its existence in 1993. The machine went through various modifications until two lines were produced, i.e., Božena 4 and Božena 5 (Figure 1). Božena 5 is controlled remotely for the necessity of high operator safety, and the mines are activated by hitting flails connected by chains to a rotating cylinder [8].

The machine is primarily intended for work in difficult and complex terrain. That is why it is equipped with a tracked undercarriage. Due to the greatest possible adaptability and optimal driving in various terrains, it is possible to convert this chassis into a wheeled one (without structural interventions, only with the replacement of the necessary structural units). The construction of the machine includes a main tool carrier which can be equipped with, for example, a milling cutter or a picker (cutting width 2655 mm and effective depth 300–350 mm). The Božena 5 also has a tool holder on the back. Here it is possible to attach additional devices such as a hydraulic arm for handling unexploded ammunition. The range of the remote control is 4500 m [9].

The construction of the machine was presented in article [7] to a reasonable extent (sensitive information). Information about the materials used for its production was also published [10]. The manufacturer discovered that the intrusion of various objects (stones, branches, sand) of an unspecified size between the belt and the rosette can cause tearing or inelastic deformation of the belt. The way to avoid this undesirable situation was indicated in [6,7,10], i.e., replacing the original S500MC belt material with S960QL material. Replacing the original material is a logical way to solve similar problems in various areas, e.g., [11,12,13] and many others. Of course, just changing the material without understanding the deeper context is not appropriate. Therefore, the intensification of the life of the belt lies in two separate solutions. The first solution is to change the material from which the belt is made, and the second (structural) is to change the location of the front wheel of the machine.

**Figure 2:**
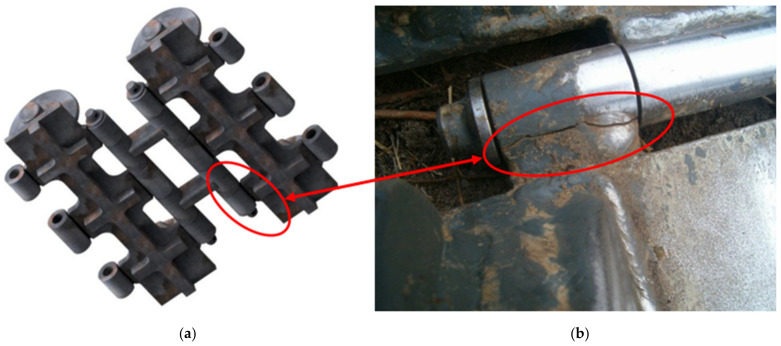
(**a**) Belt link article with damage; (**b**) The detail of its typical damage in service.

Extensive research has already been carried out [6,7,10], focusing on the determination and quantification of parameters affecting the life of a belt made of replacement material (S960QL) and its welds by progressive welding technologies (electron, laser). From there, it turned out that the belt of the machine is, among other things, stressed by fatigue loads. The current work aims to expand the research on fatigue life by using the results of the originally used material (S500MC—welded by MAG technology) and the S960QL material welded in the same way. By discussing all the results, it will be possible to unequivocally confirm the positive impact of the proposed material change. The belt of the machine is a welded component, damage to which, according to the manufacturer, is critical in the area of the weld (Figure 2).

The issue of the fatigue of welded joints is treated very conservatively in the International Institute of Welding [14]; namely, that the fatigue life of welded structures is independent of the strength limit of the material, and thus the implementation of high-strength steels does not improve the fatigue life of the welded structure. On the contrary, it assumes that the greater the yield strength of the material, the more sensitive its fatigue strength becomes to the presence of notches or to the surface condition of the material. In the welds, there are always notches related to the change in the cross-section of the weld joint. These findings then lead to the conclusion that high-strength steel welds are considered susceptible to material fatigue. The general validity of this rule was not confirmed in work [6], where research was conducted on the uniaxial fatigue life of bending and twisting loads for the base material S960QL and its welds using progressive electron and laser beam technologies. It will be very interesting and valuable to compare the results for conventional MAG technology of S500MC and S960QL materials.

In general, several fatigue analyses have been developed for welded joints, and there is a large amount of literature related to fatigue testing and the development of approaches to consider all parameters that affect fatigue [15,16,17,18,19,20]. Individual approaches are divided according to the extent to which “locality” stresses or deformations caused by external loads are defined. Then the approaches could be divided according to the method of assessment of stress (deformation) into global, structural and local. However, other classifications divide according to the stress and deformation approach as well as the fracture mechanic’s approach. All different approaches can therefore be classified according to the parameters used to describe the fatigue life or fatigue strength, for example, according to the nominal stress. This approach uses a nominal stress range determined in simple cases by equations from the theory of elasticity and strength using external or internal loading (e.g., forces and bending moments) and associated cross-sectional quantities (content of cross-sectional area or cross-sectional moduli) [21,22]. In more complex cases (e.g., statically indeterminate structures or structures that contain macro geometric discontinuities for which there is no analytical solution), FEM analysis is used—in this case, it is a modified nominal stress. The nominal stress does not consider the stress concentrators caused by the weld itself. This approach is based on extensive tests of welded joints, while there are several classification systems based on the nominal stress approach, e.g., “British Standard Fatigue Design and Assessment of Steel Structures BS 7608: 1993” or the recommendations of the International Institute of Welding “International Institute of Welding (IIW): Recommendations for Fatigue Design of Welded Joints and Components”. In these classification systems, S-N curves (Wohler curves) are divided according to the type of weld, its load and shape. The designer then only needs to determine the nominal stress and select the weld class, the so-called FAT class, i.e., the characteristic calculated from the mean values with a 95% probability of survival (5% probability of failure) based on a two-sided 75% confidence interval. The currently recommended curve for a welded joint stressed by normal stress (where bending is the dominant way of loading the belt) is FAT 71, and the highest recommended curve, which, however, is primarily intended for areas of the structure without welding, is FAT 160. It will be valuable to monitor whether the measured points (for the basic material as well as for the welded material) are located above these curves. For welded joints under shear stress, the IIW recommends only two curves, the lower of which is FAT 100 [14].

The application of high-strength steels in structures is regulated within Europe by the European Union standard Eurocode 3: Design of Steel Structures. However, the regulations are only for steels up to class S700, and the International Institute of Welding still does not give high-strength steels any advantages in terms of the fatigue life of their welded joints compared to ordinary steels. Eurocode 3 part 1–12 regulates the use of steels up to class S700, but does not contain enough experimental data and information on the characteristics and properties of structural joints in these steels [23]. Experimental data for fatigue life research were obtained using a unique testing device (the work of the authors’ workplace). The issue of the development, construction and testing methodology of the test equipment is only partially described in works [21,22,24,25]. Therefore, for its overall understanding, it is necessary in the current work to provide an additional description together with the issue of production and numerical modeling of test samples.

**Figure 3:**
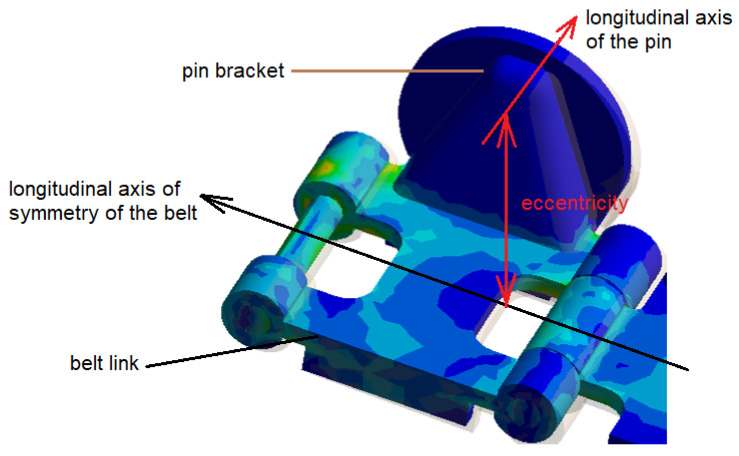
Perspective of the spatial arrangement of the belt member.

The solution to the problem from a structural point of view was based on the analysis of the process of inclusion of a foreign body between the belt–rosette pair. It turns out that the belt geometry increases its internal load. The rosette engages with the pin, which is placed on its holder and is connected to the belt link by welding joints (Figure 3).

The belt pin and the belt’s longitudinal axis of symmetry have a fixed distance (eccentricity) due to their rigid geometry (with correct guidance, i.e., without the presence of a foreign element). When designing the belt, its influence on the variability of this geometry was not taken into account in the original solution. The increasing eccentricity of the belt pin from the longitudinal axis of the belt (due to the influence of a foreign element between the belt and the rosette) will cause an additional bending moment and this will affect the value of the tension with the possibility of disabling the belt. The behavior of such an object between the wheel and the belt is stochastic. Therefore, quantifying the worst case (geometry) through simulation is difficult. Changing the strength of the material is the solution to this condition. Under these conditions, it will be appropriate for research purposes to carry out a numerical calculation of the distribution of equivalent stresses. It will be necessary to consider a structural intervention to change the geometry of the belt guide from the extreme positions, closer to the longitudinal axis of the belt, or to propose a change in the belt tensioning method and its regulation. The last-mentioned solution is probably the most suitable. The Boźena 5 has belt tensioning by means of a screw mechanism. This mechanism, due to its self-locking, does not respond to changes in internal loads (increase in dominant bending moments) caused by changes in the geometry of the belt–rosette connection. The solution to this problem will be a patented structural design of the front wheel of the machine using a linear guide in the direction of travel. The dimensions for the specific case of the machine were based on the functional calculation of the driving force on the belt provided by the TATRA internal combustion engine (TATRA Truck, Kopřivnice, Czech Republic) with a maximum power of P_max_ = 270 kW at a speed of n = 30 rpm^−1^. It is possible to disclose that of the maximum power, P_z_ = 16 kW is primarily used to drive additional equipment. The remaining P_max_ − P_z_ = 254 kW are intended precisely to ensure the driving force. This power is transmitted in Božena 5 to two hydraulic pumps marked A4VG/32 152 from the manufacturer Bosch [26].

In addition to a reliable product, the scientific contribution of this research will also be the knowledge of how to construct using high-strength steels, and the inclusion of this knowledge in safe standards and rules.

## 2. Materials and Methods

As mentioned earlier, there are two tested materials:

- Original material, steel S500MC (1.0984), used for the production of the demining machine belt, i.e., carbon, microalloyed steel with properties suitable for cold forming. The products are mainly used in the production of beams, roller wheels, drive shaft tubes, car axle covers and other structures;
- To increase the life of the belt material, it was proposed to replace the original material with S960QL. It is a high-strength structural steel hot-rolled in a refined state (hardened and tempered) (Q) which preserves properties at low temperatures (L) and guarantees compliance with the requirements of the EN 10025-6/S960QL standard. A detailed material description and microstructure were given in papers [7,10].

There, steps such as the design and production of test samples from the tested material and its welds (electron, laser, MAG) were implemented for the following:

- Cyclic bending stress (results presented in paper [7] for the base material S960QL and its laser and electron welds). The current work extends the given research by measuring the fatigue life for S960QL and S500MC materials MAG-welded and loaded by cyclic bending;
- Cyclic stress (results presented in work [7] for the base material S960QL and its laser and electron welds). The current work extends the given research by measuring the fatigue life for S960QL and S500MC materials welded by MAG and loaded with cyclic torsion;
- Static tensile tests (results for base material, laser, electron and MAG weld presented in work [10]);
- Metallographic analyzes of the base material and all tested welding technologies (electron, laser, MAG) [10];
- Measurement of material hardness and all welding technologies [10];
- Measurements of residual stresses (results for laser and electron welding presented in work [6]).

This work further extends the solution to the problem both by the methodology of creating a finite-element model of a fatigue test specimen to obtain the state of tension and deformation at its critical point, and also by strength analysis of the contact of the belt with the co-engaging gear wheel–rosette. Finally, the structural design of the patented belt tensioning mechanism supported by analytical functional calculation is carried out.

### 2.1. Tested Material

Table 1 indicates the analyzed chemical composition of the tested materials [10].

Table 2 indicates the selected analyzed mechanical properties of the given tested materials [6,7,10].

Table 3 provides basic information about the welding parameters of individual welding technologies [6].

Welding by the MAG method was performed automatically by the OTC DAIHEN welding robot (OTC DAIHEN EUROPE GmbH, Mönchengladbach, Germany), using additional wire: OK AristoRod 69; Ø 1.2 mm and protective atmosphere: M21 (82% Ar; 18% CO_2_). Laser welding was realized by a YLS 5000-S1 (ALLTEC Angewandte Laserlicht Technologie GmbH, Alsbach-Hähnlein, Germany) fiber solid laser and electron beam welding using the PZ EZ JS30 JUMBO welding complex. Tensile tests were performed on an INOVA hydraulic universal testing machine according to the ISO 6892 standard [27].

### 2.2. Experimental Device for Measuring Fatigue of Materials

**Figure 4:**
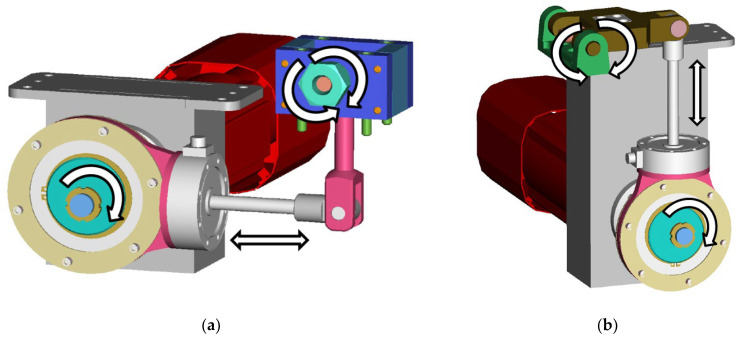
Defining the movement possibilities of individual loading mechanisms of test specimens for (**a**) torsion; (**b**) bending.

The test device is the original design of the authors’ workplace. It contains mechanisms (Figure 4) ensuring cyclic loading of the samples by torsion and bending (using an eccentric pair with fine grooving through 37 teeth, i.e., 18 load positions, see [21]).

**Figure 5:**
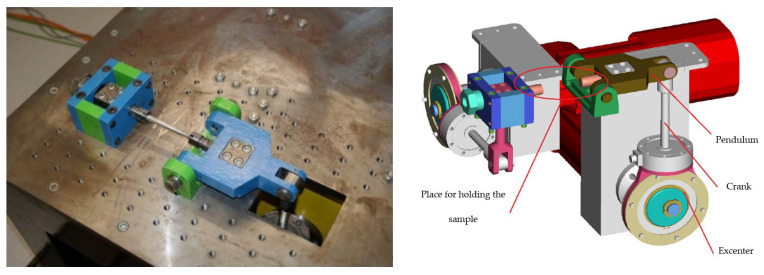
Testing device for measuring the fatigue life of materials.

The load mechanisms (Figure 5 right) are driven by synchronous servomotors controlled by the control unit via a connected desktop computer. The program enables their management both individually and simultaneously. This means that the device is capable of generating a sample load, e.g., only by twisting—then the motor is active together with the twisting mechanism and the second mechanism does not move. It also applies reciprocally, i.e., active motor and mechanism for bending and the other mechanism does not move. The advantage of the device lies in the simultaneous operation of both motors in phase or with an adjustable phase shift—loading the sample with multiaxial fatigue with a combination of bending and twisting.

**Figure 6:**
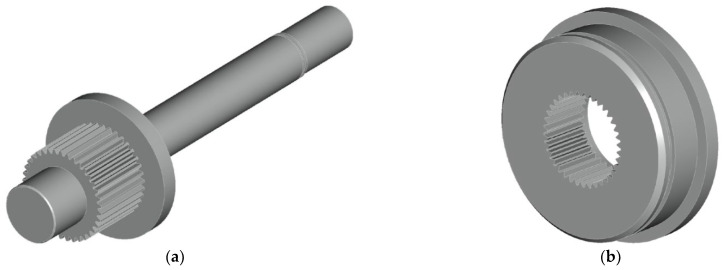
CAD model of (**a**) eccentric; (**b**) body creating an eccentric pair.

The issue of the development, construction and testing methodology of the test equipment is described in detail in the papers [21,22,23,24,25]. To expand the information about the measuring device, it can be said (Figure 5 on the right) that the rotary movements of the servomotors are transmitted through fixed shaft couplings to the eccentrics pressed into the bearings. The torque is further transmitted to the crank, which performs rectilinear movement. The latter subsequently causes bending (or twisting) of the sample, which is fixed at one end in a collet on the rocker arm and is fixed firmly (with uniaxial loading method) on the other end. The controlled quantity of the load is then the amplitude of the total deformation. The load value is set mechanically by mutually rotating the eccentric relative to the body of the eccentric (Figure 6).

**Figure 7:**
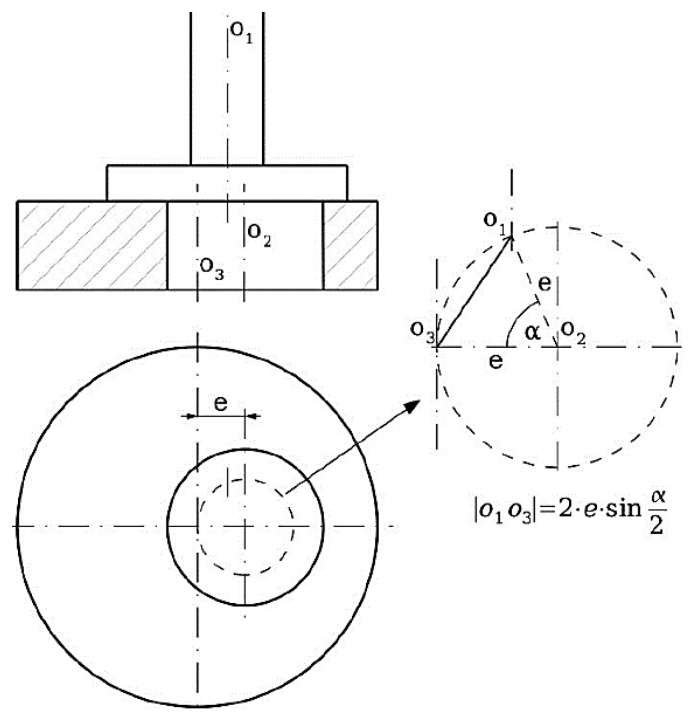
Calculation scheme of the resulting deflection of the eccentric pair.

Fine grooving is used on the eccentric pair. Such a design will ensure reliable connection and transmission of power, and the smooth and precise regulation of the load in a mechanical form. Each eccentric pair has thirty-seven positions (teeth). One position is the so-called zero, followed by eighteen gradually increasing load positions until the maximum, and then the deflections begin to decrease and repeat back to zero. This means that eighteen different deflection values can be set in this way. Three types of eccentric pairs are available (with eccentricities of 1, 2 and 4 mm). The maximum deflection is achieved by adding the individual eccentricities of both bodies. The total maximum deflection is achieved by connecting an eccentric pair with the same eccentricity, which provides options of 2, 4 and 8 mm of maximum deflection. The resulting deviation during individual rotations of the eccentric relative to the body of the eccentric can be written algebraically by Equation (1) obtained from the diagram in Figure 7:

(1) $$\left|O_{1}O_{3}\right|=2\cdots e\cdots \sin\frac{\alpha }{2}$$

**Figure 8:**
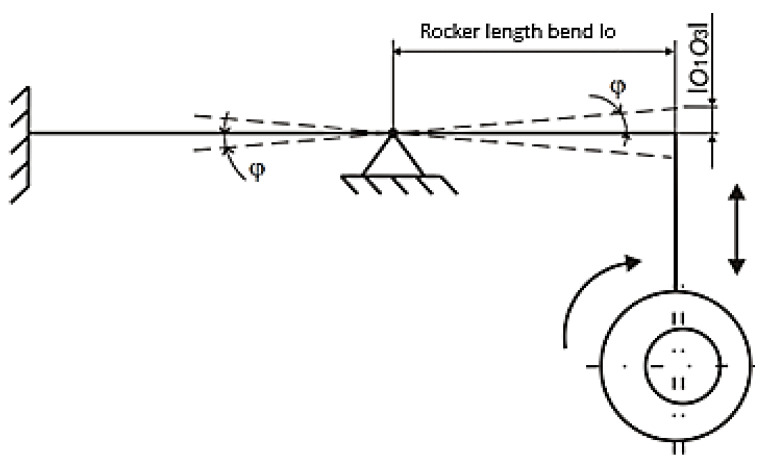
Calculation scheme of the mechanism causing bending of the test samples.

Figure 7 shows that the instantaneous deflection value is equivalent to the distance between the axis of the eccentric shaft O_1_ and the axis of the eccentric body shaft O_3_. An eccentric pair has a common axis marked O_2_ at every moment of their connection. The corresponding bending load of the samples was further expressed in the form of the collet deflection angle *φ_O_*. The value of this quantity results from the instantaneous deflection of the eccentric pair according to the diagram in Figure 8 and is given by Equation (2):

(2) $$\varphi_{O(K)}=\mathrm{arct}\mathrm{an}\frac{\left|O_{1}O_{3}\right|}{l_{O(K)}}$$

**Figure 9:**
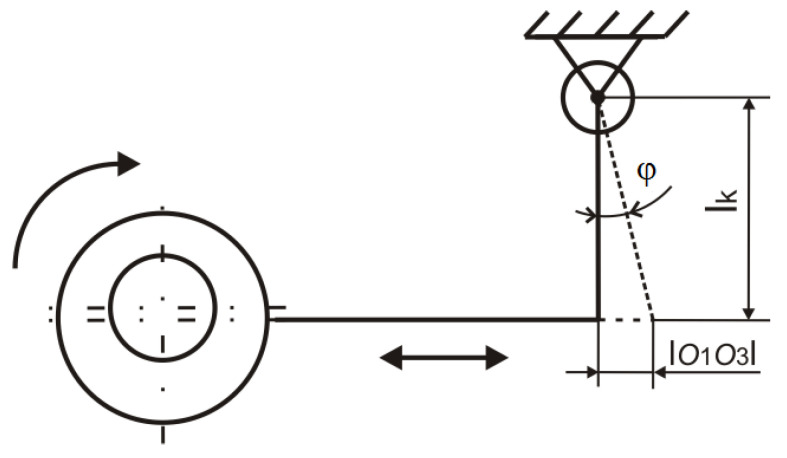
Calculation scheme of the mechanism causing torsion of the test samples.

The corresponding load of the samples in torsion was expressed in the form of the angle of twist of the sample *φ_k_*. The value of this quantity results from the instantaneous deflection of the eccentric pair according to the diagram in Figure 9 and is given by Equation (3):

(3) $$\varphi_{k}=\mathrm{arct}\mathrm{an}\frac{\left|O_{1}O_{3}\right|}{l_{k}}$$

**Figure 10:**
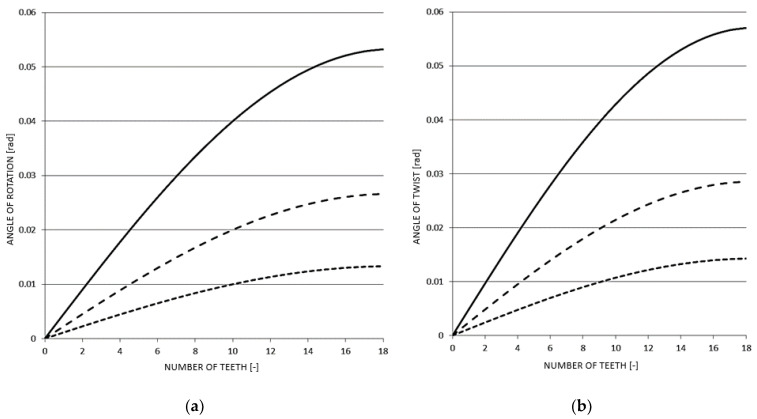
(**a**) Determined angles of rotation; (**b**) Determined angles of twist of test samples for individual eccentric pairs.

The achieved deflection angles or the twists of the samples depending on the individual positions of the eccentric pairs are shown in Figure 10.

During the fatigue tests, the torsion testing device mechanism was equipped with an eccentric pair with an eccentricity of 4 mm (Table 4) and the bending mechanism was equipped with a pair with an eccentricity of 2 mm (Table 5).

The selection of these pairs (eccentricities) was based on the results of FEM calculations in the ADINA program, which is presented in the next chapter. By comparing these results with the simulation of the stress distribution of the contact of the rosette with the belt during operation, it was found that the range of the stresses in the belt and the stress of the test samples correspond best.

### 2.3. Preparation of Test Samples for Numerical Simulations and Experimental Fatigue Measurement

**Figure 11:**
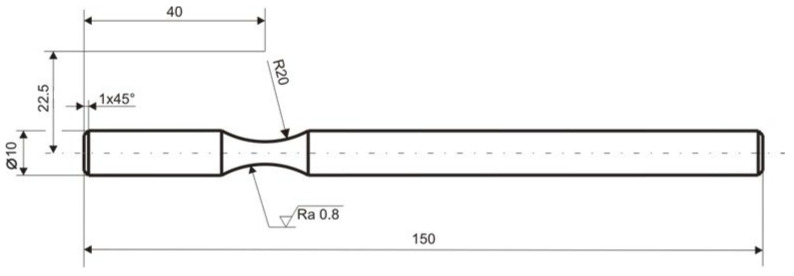
The optimal shape of the fatigue test specimen for the test condition of the experimental workplace.

The conceptual design of the shape and dimensions of the test samples was based on their simple clamping into the original testing device (Figure 4 and Figure 5). The optimal and therefore final shape of the test specimen for experimental fatigue measurement (Figure 11) was achieved by applying the results of the FEM analysis of the stress distribution during its loading [21]. The location of the neck was chosen on the basis of calculations, namely the torque and bending moment [22].

**Figure 12:**
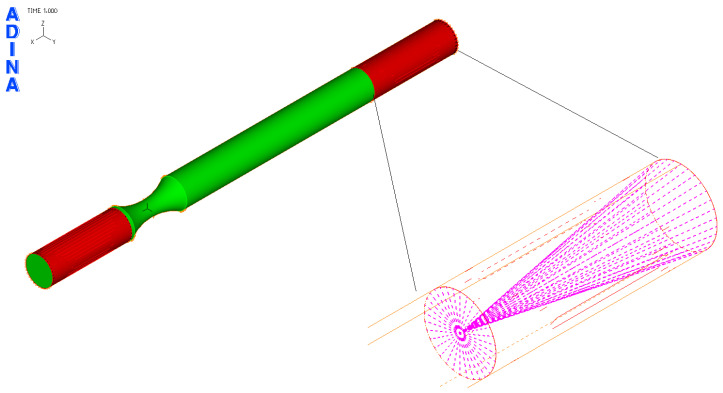
Defining groups of finite elements in the FEM model of the test specimen.

Based on Figure 11, the FEM model of the sample was created in the ADINA software 2020 (ADINA R & D, Inc., Watertown, MA, USA) (Figure 12). For a precise simulation corresponding to the real situation, it was necessary to create three “element groups”. Each of them was used to mesh a certain part of the sample.

**Figure 13:**
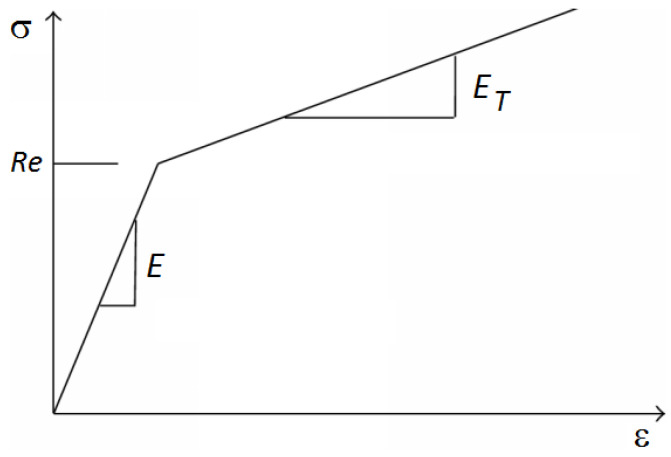
Plastic bilinear material model.

For the green part of the sample (in Figure 12), elements of the “3D-solid” type (volumetric elements) were used. The red part of the sample was created in order to precisely simulate the holding of the samples in the clamps of the testing device (shell elements were used). The last group of elements with pink color was “beam” (beam elements). These were used in order to transfer the load and boundary conditions from the place of their operation to the sample through the clamps. Material models correspond to the real properties of the materials used. These resulted from the performed static tensile tests in work [10]. Therefore, a plastic bilinear material model was used (Figure 13). Here, it can be seen that the curve beyond the ultimate strength is increasing rather than decreasing (the resulting tensile diagrams show engineering rather than actual stresses). Therefore, a plastic bilinear material model with isotropic strengthening was applied. Input data to the model and their values are listed in Table 6.

**Figure 14:**
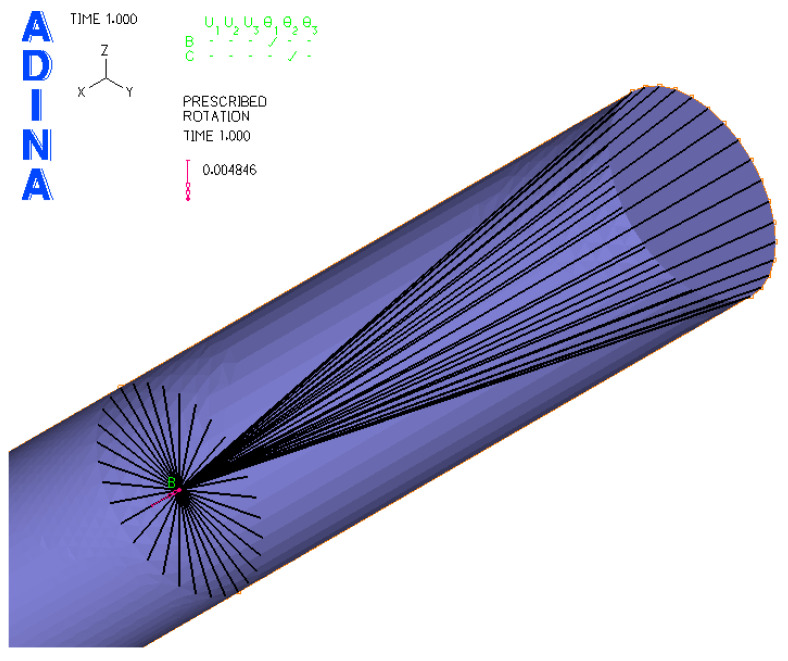
Entered boundary conditions of numerical simulation (B point—a point for a definition of boundary conditions).

In the software menu (boundary conditions section), “all degrees of freedom removed” was entered at one end of the sample (closer to the neck) in the form of possible movement except rotation around the y-axis. This corresponds to possible bending of the sample. At the other end of the sample, all degrees of freedom except rotation about the x-axis were removed. This corresponds to possible twisting of the sample. In this way, the model can be used to stress the sample by bending, twisting or their combination. The degrees of freedom were taken at the point connected to the clamps using beam elements (Figure 14).

At the same points, the unit load was defined in the form of “load displacement” by rotation around the x and y axes for bending and twisting loads. The magnitude of the load was further determined by assigning a time function separately for the bending load and separately for the torsional load. The time function used was sinusoidal for both types of load. The maximum values were entered based on the calculated values listed in Table 4 and Table 5. Calibration curves were determined for the tested materials by numerical analysis using the FEM program Adina. Their function was to clearly display the dependence between the deformation amplitude (achieved by the test device) and the distribution of the equivalent stress in the test sample. The numerical values of the results of these simulations are given in Table 7, Table 8, Table 9, Table 10, Table 11, Table 12, Table 13 and Table 14.

Due to the very similar tensile diagrams of the base material S960QL and its welds by progressive electron and laser technologies [10], the values shown in Table 7 and Table 8 are common.

### 2.4. Fatigue Life Measurement

Testing of the specimens was performed in the controlled strain amplitude (Manson–Coffin) as allowed by the testing equipment (Figure 4 and Figure 5). During the loading in the form of torsion, the controlled quantity was the total amplitude of the shear deformation, i.e., the slope γ_xy_. The bending load was defined by the controlled quantity of the total proportional deformation amplitude ε_xx_. Loading was carried out with a constant zero-mean strain amplitude with a symmetrical (cycle asymmetry factor R = −1 [-]) sinusoidal cycle with a loading frequency of 35 Hz. The temperature in the laboratory varied between 20 and 23 °C. The recorded number of cycles corresponded to the lifetime of individual samples until fracture. In the case of lifetimes higher than 10^7^ cycles, the tests were interrupted.

It should be emphasized again that during the experiments, the mechanism of the testing device for torsion was equipped with an eccentric pair with an eccentricity of 4 mm (Table 4) and the mechanism for bending was equipped with a pair with an eccentricity of 2 mm (Table 5). The selection of these pairs was made based on FEM calculations in the ADINA program. It was found that the range of stresses achieved at individual load levels for the given eccentric pairs best corresponded to the load area of the belt material of the demining machine during its operation. Cycle count records for the base material, welding technologies and loading methods will be shown in tables. The data will be processed in the form of Manson–Coffin curves with the indicated coefficients of determination R^2^ of the power regression model translated by the data. The data will also be plotted in the following form: amplitude of shear and normal stress as a function of the number of cycles to fracture.

### 2.5. Functional Calculations Accompanying the Design Proposal for Changing the Belt Tension of the Božena 5 Machine

When designing structural units of transport and handling equipment, it must be ensured that the designs are able to fulfill the “meaning of their existence” during their entire lifetime, while meeting the conditions of safety and reliability. It is essential that the designs have sufficient resistance to loads and other factors that are expected to affect the given equipment during operation, together with a sufficient degree of safety. The estimation (choice) of safety coefficients is influenced by many factors; in any case, certain experience is necessary. For example, it is recommended to be very careful when the structural units work in extraordinary conditions (tropical climate, i.e., high temperature and high relative humidity, heavy dense operation, high temperature with lower relative humidity and rough handling, continuous operation of the equipment, etc.).

As already mentioned in the Introduction, the power of the Božena 5 combustion engine is transmitted to two hydraulic pumps. The necessary parameters of the pump included in the calculation are listed in Table 15 [28].

The power of the combustion engine entering the pumps causes an increase in their output speed through the flow of liquid. This flow rate is given by Equation (4):

(4) $$q_{v}=\frac{V_{Gmax}\cdot n\cdot \eta_{p}}{1000}$$

The hydraulic fluid subsequently drives a total of four hydraulic motors marked A2FM 63 (manufacturer Bosch) [29]. The parameters of such an engine are listed in Table 16 [27].

The revolutions from the hydraulic motor further enter the wheel gearbox, and the instantaneous value of the revolutions can be described by Equation (6). The liquid flow through one pump from Equation (4) must be redistributed between two hydraulic motors (5):

(5) $$q_{\mathrm{vm}}=\frac{q_{v}}{2}$$

(6) $$n=\frac{q_{vm}\cdot 1000\cdot \eta_{p}}{V_{GMmax}}$$

The total mechanical efficiency of the hydraulic part of the performance is given by the product of the partial efficiencies of the individual members. Then it is possible to determine the power per hydraulic motor by the ratio of the power of the combustion engine to the number of hydraulic motors (7):

(7) $$P_{k}=\;\frac{P_{p}}{4}\cdot \eta_{c}$$

The torque transmitted by one hydraulic motor can be determined from the output (8):

(8) $$M_{k}=\frac{P_{k}}{2\cdot \pi \cdot \frac{n}{60}}$$

Part of the hydraulic motor is a wheel gearbox with a mechanical efficiency of 80% and a gear ratio i = 38.6. At the output of the gearbox, the torque given by (9) is then achieved:

M_kv_ = M_k_ · i · η_p_ (9)

The resulting force acting on the belt of the demining machine transmitted by a sprocket with a pitch radius of r = 0.465 m will then be (10)

(10) $$F_{p}=\frac{M_{k}}{r}$$

**Figure 15:**
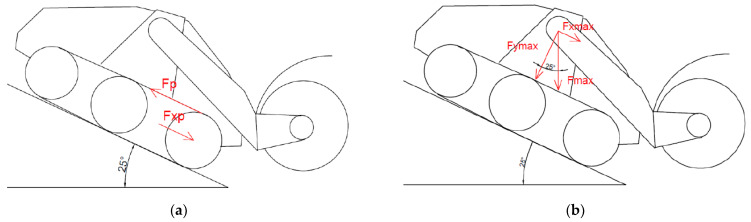
(**a**) Considered action of the driving force F_p_ on the front wheel. (**b**) The component of the gravity force in the incline of the machine.

The manufacturer indicates the maximum total vehicle weight is 13,090 kg. It is made up of the sum of the weight of the vehicle itself (10,240 kg) and the weight of the ground cutter attachment (2850 kg). In the functional calculations aimed at quantifying the forces acting in the proposed tensioning mechanism, the limit state was calculated. This corresponded to the action of the entire weight of the vehicle on the front axle (the vertical force is therefore 128,413 N). A vertical force of 64,206.5 N is thus applied to one front wheel. Furthermore, the limit state considered the creation of a horizontal force that acts on the front wheel and arises when the vehicle is backed up to a slope (the most unfavorable condition identified by the manufacturer). This generally acting force consists of two basic components. The first is the longitudinal force component, which is caused by the driving force applied to the belt. In Figure 15 on the left, the driving force F_p_ acts on the arm corresponding to the pitch diameter of the wheel. The longitudinal force will be half the size because it acts on the half arm (11):

(11) $$F_{\mathrm{xp}}=\frac{F_{p}}{2}$$

The second component is created by the weight of the vehicle in the maximum gradient (the manufacturer indicates the maximum gradient α = 25°). This component is calculated using Equation (12), which is derived from Figure 15 on the right.

F_xmax_ = F_max_ · sin α (12)

The resulting longitudinal force acting on the wheel in the direction of travel of the vehicle is relative (13). It is equal to the sum of the longitudinal force from the weight of the vehicle and the longitudinal component of the driving force acting on the vehicle belt.

F_x_ = F_1xmax_ + F_xp_ (13)

It is generally known that the belts of tracked vehicles are most stressed near the point of engagement with the chain wheel. Therefore, the highest stress values are also expected here. To quantify these values, a static numerical calculation was performed using the Ansys program. An accurate CAD model of the rosette and belt contact was modeled in its graphic environment. Material parameters were specified for the model, the values of which are given in Table 17. The boundary conditions corresponded to the real state. The load was applied in the form of the calculated belt tension force.

### 2.6. Patented Design Solution for Tensioning the Belt

Several requirements are placed on the proposed design solution:

- The design itself is necessary due to the continuous creation of the necessary pressure force between the rosette and the belt;
- It must enable the torque to be transferred from the rosette to the belt;
- It must allow easy handling when changing the belt;
- Tensioning must ensure the necessary pressure force between the belt and rosette in normal operation mode;
- In the event of a foreign object entering between the wheel and the belt, it must allow movement negating the increase in force leading to the belt tearing.

**Figure 16:**
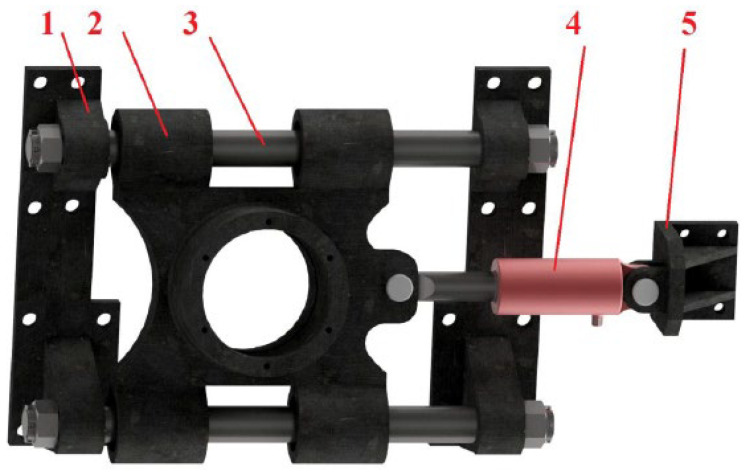
CAD model of the structural design of belt tensioning using two guide rods.

All of this is made possible by the construction solution of storage using two guide rods, as shown in Figure 16. As can be seen, the design includes guide rods (position 3) to which the wheel hub (position 2) is attached by means of a sliding bearing. The guide rods are stored in the houses (position 1) and can be attached to the vehicle, e.g., by means of screws or a welded joint. The movement of the hub sliding sleeves in the longitudinal direction of the vehicle is controlled by a hydraulic cylinder (position 4). This is how the tensioning force is derived, the opposite reaction of which is transmitted to the grip (position 5). The handle can again be attached to the vehicle using screws or a welded joint.

**Figure 17:**
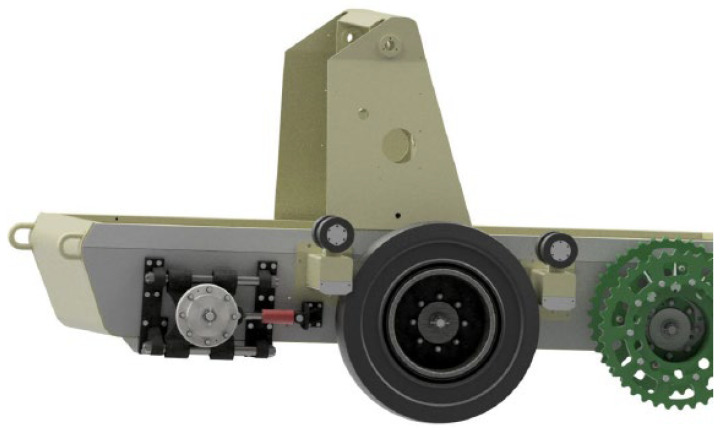
CAD model of the Božena 5 chassis with mounting of the proposed design solution for tensioning the belt.

**Figure 18:**
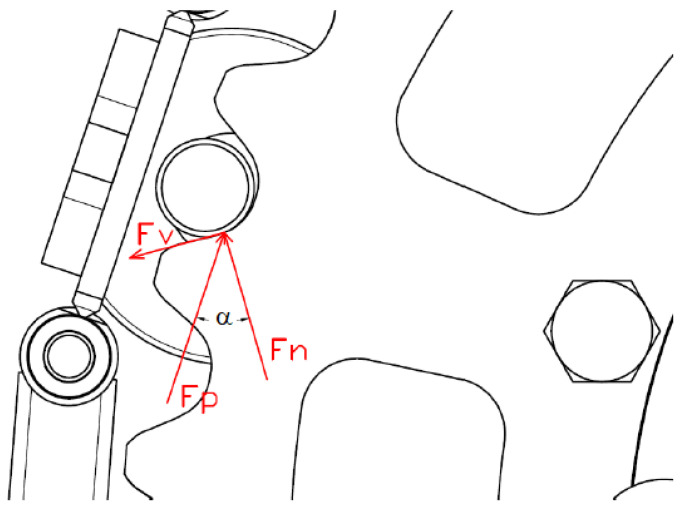
Strength ratios in contact between the belt and the rosette.

The structural design used in a real vehicle (Figure 17) requires a strength check based on accurate knowledge of the strength ratios of the belt contact with the rosette. It has been identified that the following forces act on the belt at the maximum considered load (backing up the slope):

- Driving force, divided into two components. The first of them acts perpendicular to the tooth of the rosette (normal force) and the second acts perpendicular to the normal force (tangential force). This force tends to push the belt pin out of its seat in the rosette (Figure 18). We assume a uniform distribution of the driving force in the six engaging teeth (due to the angle of the rosette belt given by the vehicle geometry);
- Centrifugal;
- Tension;
- Friction.

In that case, the structure will be loaded with a vertical force clearly determined by the functional calculation in the previous point. Its value is 64,206.5 N. However, the loading of the structure is also carried out in the longitudinal direction by the tensioning force of the belt. This needs to be determined by further calculations. The tangential force F_v_ can be determined as follows (14):

(14) $$F_{v}=\frac{F_{p}}{2}\cdot \sin\alpha$$

where α_1_ is the deviation of the normal force from the driving force (given by the geometry α_1_ = 32°). The centrifugal force of the belt can be determined as follows (15):

F_o_ = m · (2 · π · n)^2^ · R (15)

For its quantification, it is necessary to know the mass of the belt link m_b_ (kg), the revolutions n (s^−1^) and the radius of the chain wheel. The values of the individual parameters included in the calculation are listed in Table 18.

The frictional force is defined using Coulomb’s Equation (16):

F_t_ = F_n_ · f (16)

Normal Strength (17):

(17) $$F_{n}=\frac{F_{p}}{6}\cdot \cos\alpha$$

The tension force is given by the sum of the centrifugal force, the tangential force (acting against the direction of the tension force) and the friction force (acting in the direction of the tension force) (18):

F_N_ = F_o_ + F_v_ − F_t_ (18)

The tension force determined in this way corresponds to only one interlocking link. The total strength can be determined by multiplying by the number of links, i.e., F_nap_ = 6 · F_N_. Driving the machine also affects belt tension. The force F_x_ (N) acting on the belt along the vehicle causes the front wheel to move. Therefore, the clearance in the belt increases and this leads to its slipping. This phenomenon is prevented by increasing the tensioning force of the belt. The longitudinal force F_x_ and the tension force F_nap_ must therefore be added together. The resulting tension force is then F_vn_ (19):

F_vn_ = F_x_ + F_nap_ (19)

## 3. Results

Chapter 3 discusses the main results from experimental measurements, numerical simulations and analytical solutions of the presented research. In particular, the presented results focus on the following:

- Test samples for fatigue life assessment;
- Calibration curves of the tested materials S960QL and S500MC;
- Test methodologies and experiment results (fatigue);
- Computational model of the tensioning mechanism;
- Patented design solution for tensioning the demining machine belt.

### 3.1. Creation of Test Samples for Numerical Simulations and Experimental Fatigue Measurement

**Figure 19:**
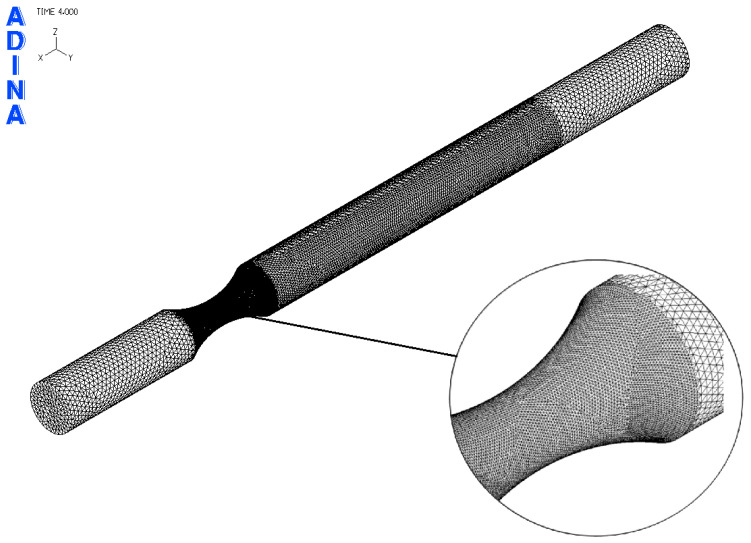
Prepared FEM model of the test specimen for numerical calculation of stresses achieved during the test process.

**Figure 20:**
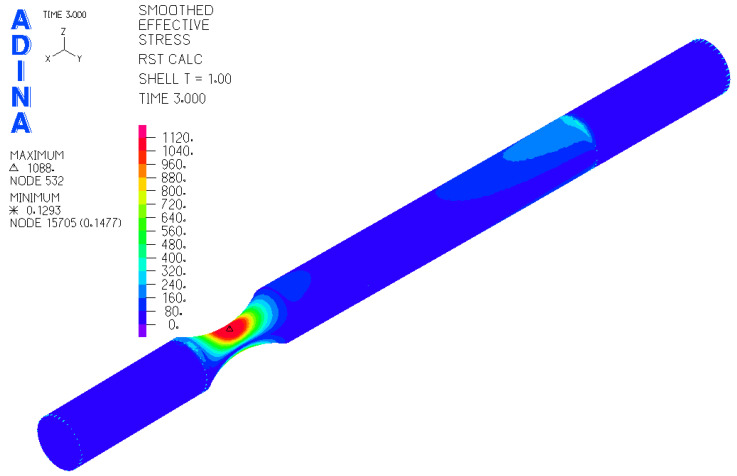
Results for the distribution of the reduced stress of the sample under bending stress.

**Figure 21:**
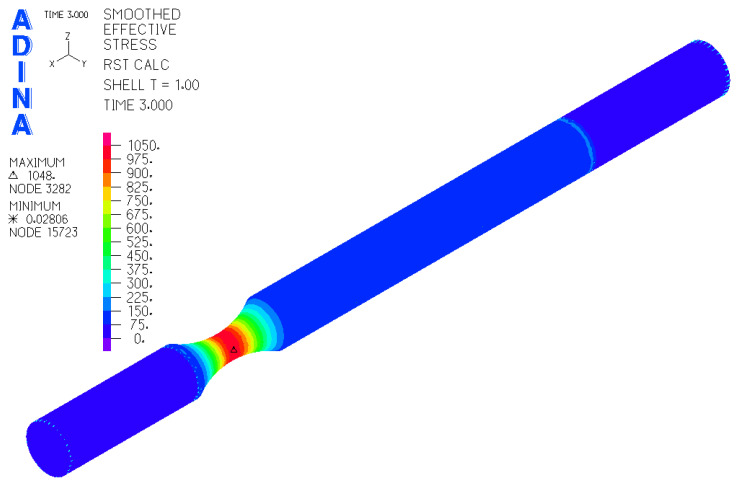
Results for the reduced stress distribution of the torsionally stressed sample.

**Figure 22:**
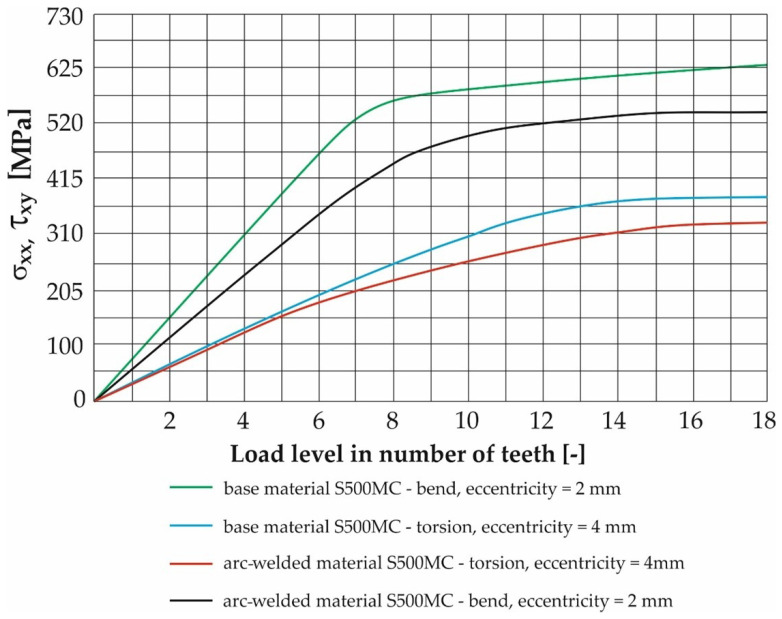
Calibration curves of values of shear and normal stresses of the material of the S500MC test sample and its arc welds for all levels of bending and twisting loads.

**Figure 23:**
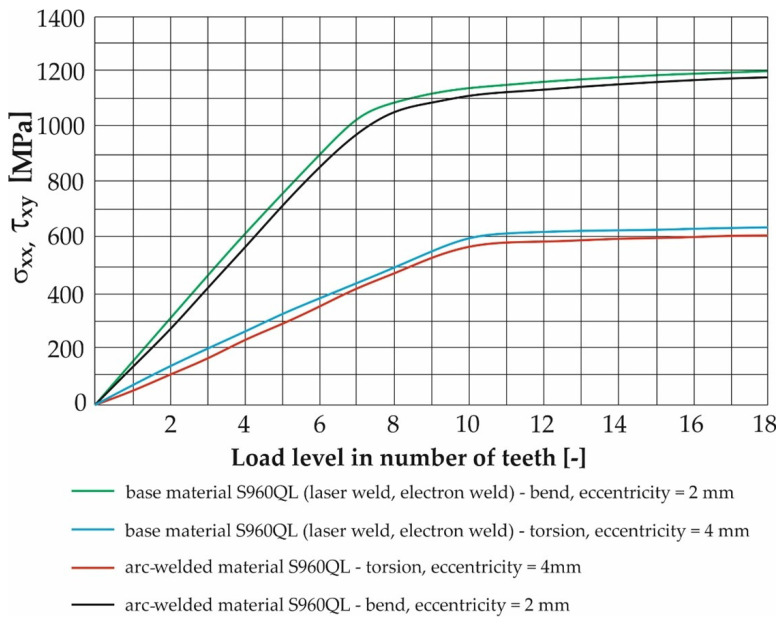
Calibration curves of values of shear and normal stresses of the material of the test sample S960QL and its welds (laser, electron, arc) for all levels of bending and twisting loads.

The result of the steps mentioned in Section 2.3 is the FEM model of the test sample for fatigue life measurement (Figure 19). Subsequently, a series of simulations was performed for each load level (mutual rotation of the teeth of the eccentric pair). For torsional loading, it was an eccentricity of 4 mm, and for bending loading, it was an eccentricity of 2 mm. As said earlier, calibration curves were determined for the tested materials by numerical analysis using the FEM program Adina. The finite element mesh is depicted in Figure 19 and the results are shown in Figure 20 and Figure 21. Their function is to clearly display the dependence between the number of teeth of the eccentric pair and the distribution of normal and shear stresses in the test sample. The curves are a transitional characteristic between the consistency of the results at the workplace (where the machine operator works better with the number of teeth of the eccentric pair) and the relevance in the context of the IIW regulations for the achieved stresses of individual load levels. The distribution of reduced bending stresses in the tested sample is shown in Figure 20. The results for the stiffness are shown in Figure 21. The images represent a graphic representation of a specific case selected from the data presented in Table 7 and Table 8. Therefore, the presence of images has a more documentary function. However, all tabular values are clearly shown in the form of calibration curves using Figure 22 and Figure 23.

### 3.2. Results of Fatigue Life Measurement of Tested Materials S960QL and S500MC Welded by MAG Technology

So far, results from this area have been presented for measuring the uniaxial fatigue life for the bending and torsion of the base material S960QL and its welds by electron and laser technologies [6]. The present work expands the field with the results of the same measurement for the MAG technology of both the original S500MC material and the proposed S960QL material. The expected difference in results will provide the possibility of analysis, discussion and optimization from the point of view of eliminating the failure rate of demining machine components. The number of measurements at one load level with good agreement of the results was three. If the results had a greater dispersion of values, then the number reached six measurements. The determined average values of the number of cycles to fracture (rounded to a whole number) for all technologies (due to the possibility of immediate comparison) are presented in Table 19, Table 20, Table 21, Table 22, Table 23, Table 24, Table 25, Table 26, Table 27, Table 28, Table 29, Table 30 and Table 31.

**Figure 24:**
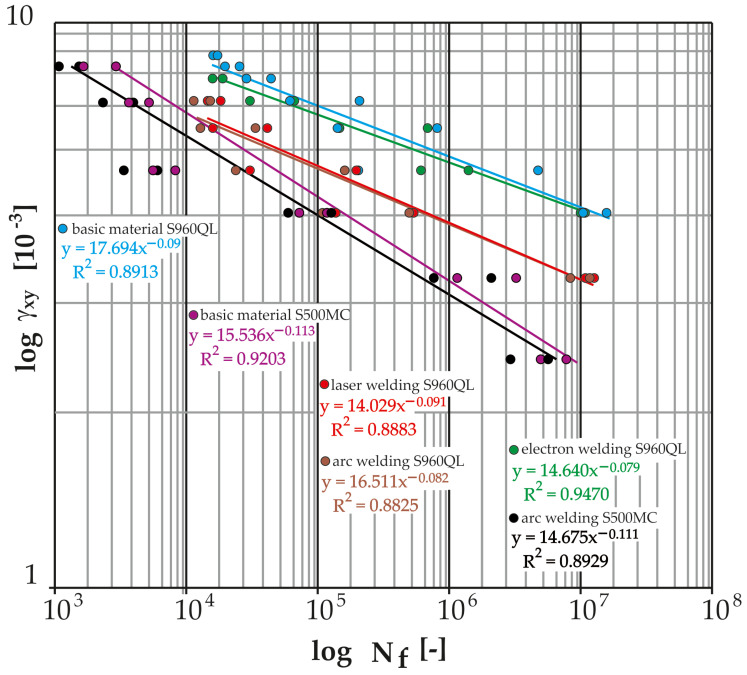
Fatigue curves (Manson–Coffin) of the tested material S960QL and its welds (electron, laser, MAG) and the tested material S500MC and its weld (MAG) from cyclic torsion loading.

**Figure 25:**
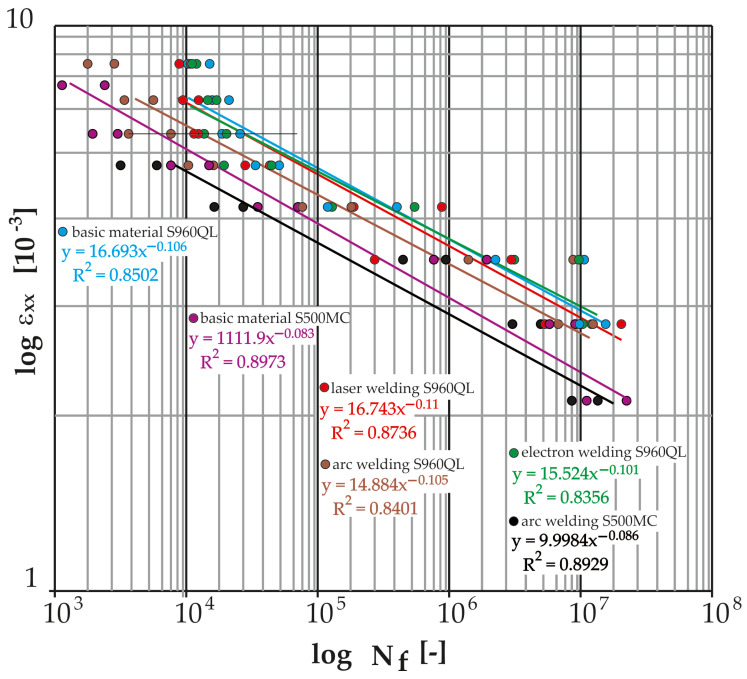
Fatigue curves (Manson–Coffin) of the tested material S960QL and its welds (electron, laser, MAG) and the tested material S500MC and its weld (MAG) from cyclic bending loading.

The measured data (Table 19, Table 20, Table 21, Table 22, Table 23, Table 24, Table 25, Table 26, Table 27, Table 28, Table 29, Table 30 and Table 31) were further processed in the form of Manson–Coffin life curves (Figure 24 and Figure 25) along with the indicated coefficients of determination of the power regression model overlaid with the data (R^2^). For the sake of clarity, the graphs show the minimum and maximum number of cycles to fracture for each weld and tested load level.

Figure 25 thus presents the measured data from the cyclic tests of the test samples (base material S960QL and its MAG welds, laser and electron and base material S500MC and its MAG weld) in logarithmic coordinates of the shear deformation amplitude versus the number of cycles to fracture. It can be observed that the base material S960QL achieves the highest curve, and its electron beam welds show only a slightly shorter lifetime. The MAG-welded S960QL material showed the shortest lifetime (compared to other S960QLs). The shortest service life was recorded for the S500MC material MAG technology used by the machine manufacturer. For the high-strength S960QL steel under shear stress, it is important to know that the welds (MAG and laser) show a relatively small decrease in service life compared to the base material. In the case of electron beam welding, this decrease is negligible.

**Figure 26:**
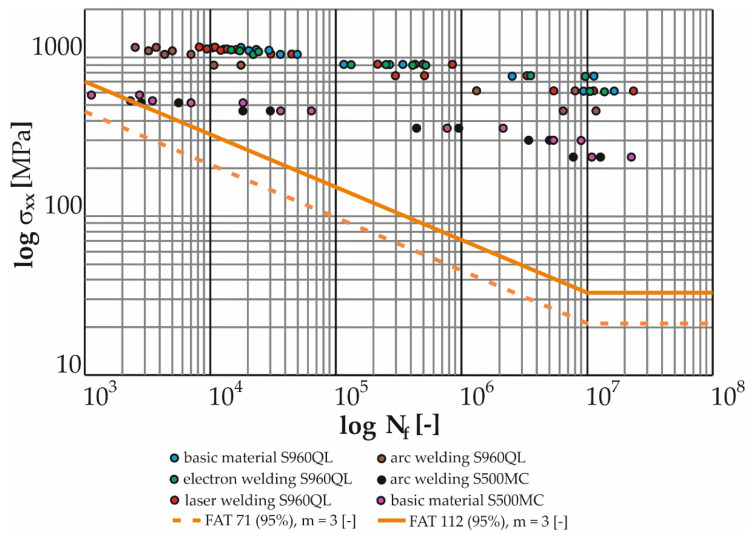
Comparison of measured data with recommended FAT curves for normal stress in the weld.

Figure 26 presents the measured data from cyclic tests of the test specimens (base material S960QL and its MAG welds, laser and electron and base material S500MC and its MAG weld) in logarithmic coordinates of the relative deformation amplitude versus the number of cycles to fracture. As in the case of torsion, as well as in the case of bending load, observation can lead to the conclusion that the base material achieves the highest curve (at the number of cycles to fracture below 10^5^). In the same interval, the curves of the laser and electron beam are approximately at the same lifetime, i.e., just below the curve of the base material S960QL. By further reducing the value of the deformation, the curve of the electron weld increases until the time when it becomes equal to the base material at the number of 10^7^ cycles. The MAG weld for S960QL again showed the shortest lifetimes across the spectrum of load levels. Here, it is important to know that due to bending stress (the dominant stress for the Božena 5 belt), welds (electron and laser) show a negligible decrease in service life compared to the base material. The service life of the S500MC base material is shorter than the service life of all S960QL welds including MAG technology. For this reason, it was expected that the welds of the S500MC material would have by far the shortest lifetime with a bending fatigue limit corresponding to approximately 235 MPa.

Regarding the suitability of using the fatigue-resistant S500MC material in machine operation, it is necessary to compare the measured data with the FAT curves according to the IIW. The highest FAT curve that the IIW allows for normal stress is FAT 160 with a slope of m = 5 [-]. This curve is used for non-welded parts of the structure. The condition for its use is the grinding of sharp edges and surface defects. For high-strength steels (S960QL), a higher (unspecified) FAT curve can also be used. Here, the essential condition is that the material must be verified by testing. The highest curve allowed for welded joints is FAT 112 with a slope of m = 3 [-], in addition to 100% NDT inspection (non-destructive testing methods). For a transversely loaded butt weld (not the case of Božena 5) welded from one side without a backing with the root of the weld inspected by NDT, the FAT 71 curve is permissible.

For welds loaded with shear loads, the IIW gives only two curves. FAT 100 is to be used for the base material or fully welded butt weld. The use of FAT 80 is recommended for partially welded butt welds or fillet welds. The FAT curve is a graphical representation of Equations (20) and (21) (Wöhler curves):

N = C/Δσ^m^ (20)

N = C/Δτ^m^ (21)

According to the IIW,

- m = 3 [-] structures (steel) stressed by normal stress up to 10^7^ cycles;
- m = 5 [-] structures (steel) stressed by shear stress up to 10^8^ cycles.

All tested samples provided data with which power regression curves were overlaid by the method of least squares. All obtained equations of regression curves (Manson–Coffin and Wöhler) are listed in Table 32 and Table 33.

Figure 26 graphically displays the measured data from cyclic bending tests for all tested samples in logarithmic coordinates of the normal stress amplitude versus the number of cycles to failure. Here, it can be seen (as well as on the Manson–Coffin curves in Figure 25) that the base material S960QL achieves the highest curve. For lower cycle numbers (10^4^), the curves of the laser- and electron-welded material are approximately at the same level and at the same time just below the curve of the base material. As the number of cycles to fracture increases, the curve for the electron-welded material increases. The trend continues until finally, at 10^7^ cycles, the curve of the electron weld becomes equal to the base material. However, in general, the difference between the measured data of the base material and the advanced technologies of the welded material (S960QL) is very small, and all measured points are located safely above the highest curve recommended by the IIW for normal stress (i.e., FAT 160 with a slope of m = 5 [-]). In the case of the S500MC material, both welded and non-welded versions, its use is possible from the point of view of fatigue loading in bending. The risk arises with low numbers of cycles to fracture (around 1000), when the service life of the welded material very quickly approaches the FAT curve. However, the Božena 5 material does not approach such a loading state under normal operating conditions.

**Figure 27:**
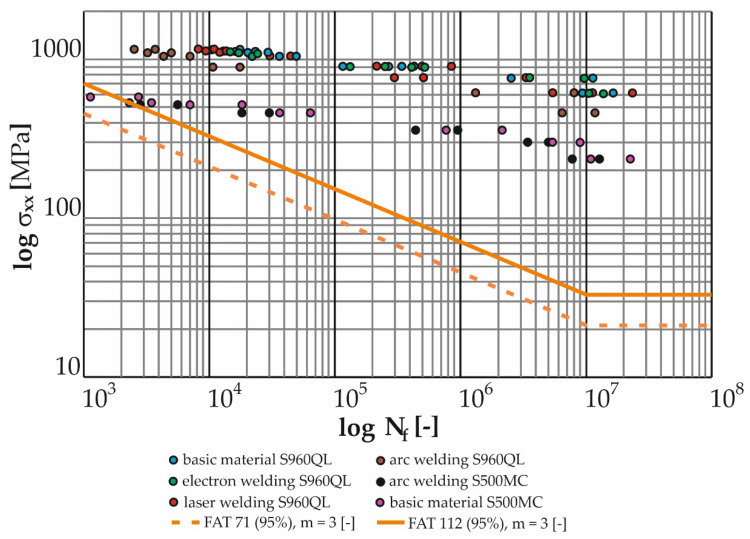
Comparison of measured data with the recommended FAT curve for tangential stress in the weld.

The same procedure was carried out for the measurement of fatigue in torsion. Figure 27 graphically shows the measured data from cyclic tests in torsion for all tested samples in logarithmic coordinates of the shear stress amplitude versus the number of cycles to failure. Here, it is possible to see (also on the Manson–Coffin curves in Figure 24) that the highest curve is reached by the base material S960QL, a little lower than the electron beam weld and laser-welded material. But an important point is that both curves belonging to the samples welded by progressive technologies show a very small decrease compared to the base material. All the measured points are well above the IIW’s recommended shear stress curve, i.e., FAT 100. The basic material of S500MC is in the whole range of measured loads in safe areas. The situation is different for arc welds of material S500MC, where the measured data correspond to the trend of the FAT curve for low numbers of cycles (up to 10^4^). In lower amplitudes of deformation (for lower loading values) then, there is an increase in the service life in comparison with the FAT curve. Again, it can be said that the Božena 5 material does not approach such a loading state under normal operating conditions. The problem can arise during overloading of the machine.

### 3.3. Analysis of the Structural Design of the Belt Tensioning Change for the Machine Božena 5

This section presents the results obtained by solving the constructed mathematical model (Equations (4)–(19)) in tabular form (Table 34).

As can be seen, the resulting tension force has a value of 49,028.52. Its effect, however, needs to be increased by the value of the axial force of the return spring with a value of 5000 N. It is also necessary to introduce a certain reserve in case the machine encounters an unpredictable situation requiring a further increase in tension force. Therefore, the resulting tension force with a value of 60,000 N was chosen and the hydraulic system was designed for this value. The hydraulic cylinder that will provide this force is single-acting with a piston diameter of d = 70 mm and a stroke of l = 150 mm. With a single-acting cylinder, the displacement of the piston is from one side with the help of hydraulic fluid pressure, and from the other side, a spring acts, which returns the piston to its initial position. It will be necessary to calculate the necessary control pressure and propose the operation of the hydraulic belt tensioning control system.

### 3.4. Numerical Simulation of a Tensioning Mechanism Made by Using Guide Rods

**Figure 28:**
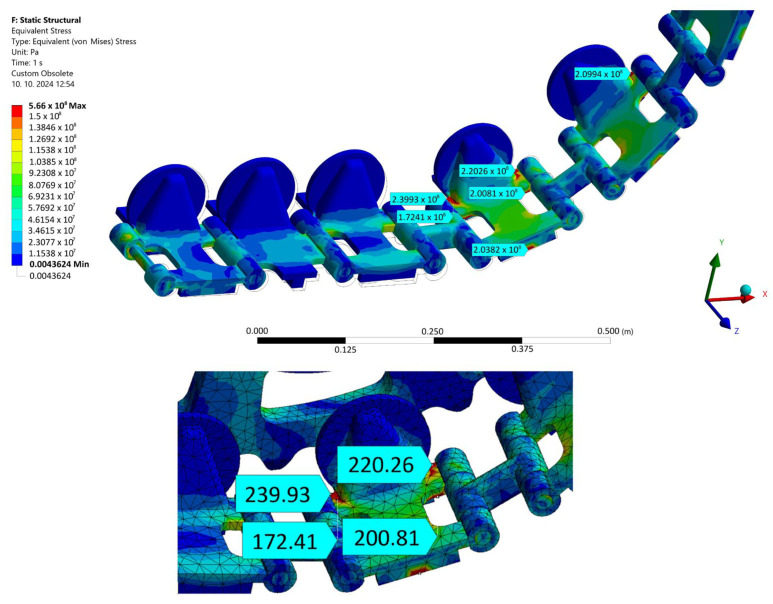
Equivalent von Mises stress in the belt–rosette structure at the maximum tension force F_vn_ = 60,000 N allowed by the tensioning system.

A strength analysis of the contact between the rosette and the belt was performed using Ansys. In its graphic environment, individual components were modeled using volume elements. The material parameters used are presented in Table 6. The boundary condition of the load corresponded to the torque transmitted by the rosette M_kv_ = 10,483.76 Nm through the belt tension force F_vn_ = 60,000 N through the wheel hub. The boundary condition chosen in this way corresponded with the real state. Linear tetrahedron elements with an element size of 10 mm were used for meshing. The result of the simulation can be seen in Figure 28. The examined belt was most stressed near the engagement with the chain wheel and in the most unfavorable part of the structure—in the weld joint. At the same time, this is where the manufacturer-identified violation occured (Figure 2, right). The stress value in this part reached approximately 240 MPa. In other parts, the tension was significantly lower. It is not assumed that this maximum load could cause damage to the base material of the belt by itself, even in its “weaker” version in the form of S500MC (EN 1.0984). However, the place and way of loading is critical. As seen in Figure 2 on the right, the place with the greatest load concentration is distributed in the weld joint.

In works [6,9], it is stated that the fracture surface did not correspond to the process of fatigue crack propagation, which corresponds to the calculated value of the maximum load that did not reach such values. By using values from Figure 24, in the case of S960QL steel, the load level set by turning the eccentric pair by two teeth corresponds to a stress of about 240 MPa. At such a low deformation amplitude, the measurement was no longer carried out, because even at the lowest tested level corresponding to five teeth, the average lifetime for the MAG weld was more than 3.5 million cycles. For this reason, it can be said that, from a theoretical point of view, the use of the original S500MC material is suitable under the conditions in question.

**Figure 29:**
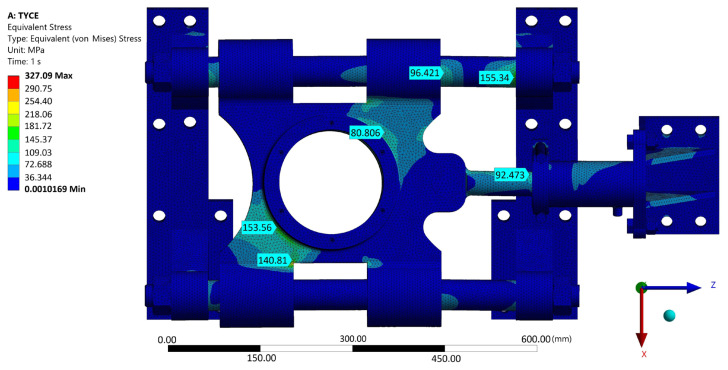
Values of the reduced tension of the proposed design of the tensioning mechanism of the belt when combining the maximum possible force effects.

**Figure 30:**
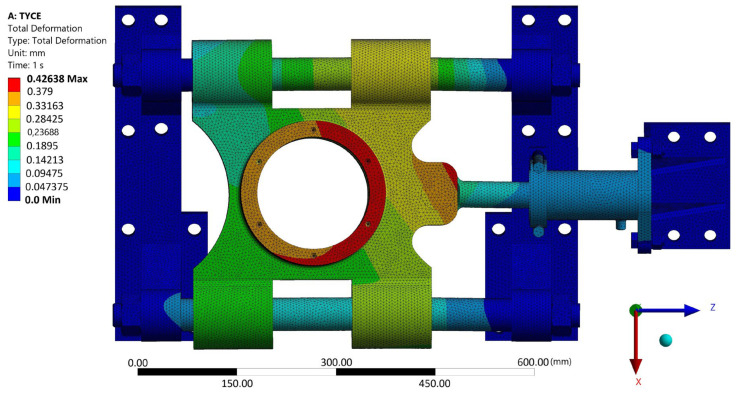
Displacement values of the proposed design of the belt tensioning mechanism when combining the maximum possible force effects.

**Figure 31:**
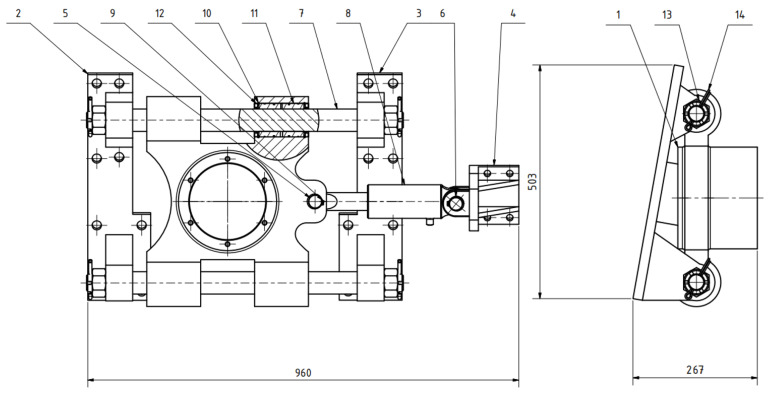
Outline and side view of the tensioning mechanism (1—wheel hub, 2—front bracket, 3—rear bracket, 4—hydraulic cylinder bracket, 5—pin 1, 6—pin 2, 7—guide rod, 8—hydraulic cylinder 70/30/150, 9—retaining ring 30, 10—retaining ring 75, 11—slip sleeve, 12—bearing 50 × 75 × 8, 13—crown nut M36, 14—cotter pin).

Next, a strength simulation of the structural solution model was carried out in the Ansys program (from Figure 16 and Figure 17). The model was assigned a load in the form of a calculated vertical force with a value of 64,206.5 N (see Section 2.5). In the longitudinal direction, the structure was loaded with a belt tension force (60,000 N). Structural steel S235 with a yield strength of 235 MPa can be used as a material. Other material properties entered into the program correspond to Table 2 and Table 6. Figure 29 shows that the highest stress values reached are approximately 150 MPa. It is possible to conclude, based on the material used, that this structure can withstand the given stress. However, it is also important to check the displacements of the structure. As can be seen in Figure 30, it is not assumed that 0.44 mm as the maximum displacement value will have a fundamental impact on the functionality of the given technical solution. However, this would not be the case in the case of a fixed attachment of the hydraulic cylinder presented in Figure 29 and Figure 30. Displacement of the wheel hub could cause deformation of and stress to the hydraulic cylinder. Therefore, the final design of its attachment is shown in Figure 16. Here, it can be seen that the bearing of the cylinder is pivoted by means of pins located on both sides of the cylinder. This ensures the minimization of resistance in the event of its deviation. Figure 31 shows an outline and side view of the tensioning mechanism.

## 4. Assessments

Results and findings from previous extensive research [6,7,10] were also implemented in the evaluation. This step ensured optimal conditions, i.e., solving the problem of belt damage in operation. The damage was observed mainly in the reversed driving mode on sandy ground or during the phenomenon of inclusion of a foreign element between the kinematic pair belt–rosette. It goes without saying that such a condition significantly affects the service life and competitiveness of the machine. The original version of the S500MC steel belt (EN 1.0984) was subjected to a detailed analysis. It can be said that this steel is suitable for welding with the use of conventional technologies, where the MAG used by the manufacturer can also be included. From the analysis of research works, it can be said that with the increasing thickness and strength of products made of this steel, the risk of cold cracks also increases [30,31,32,33]. The cause of their formation was explained by the diffusion of hydrogen into the weld metal during welding, which is caused by the lack of time to stay in the transition state from the melt to the solid state [10]. In the cases of using conventional welding technologies, the microstructure of S500MC steel had a columnar structure oriented in the direction of the temperature gradient with a significant build-up limit, when the temperature field during welding was strong enough to cause austenite grain growth. Furthermore, it was possible to observe that the grain size decreased with increasing distance from the weld.

It is true that in the searched research works, it is extremely difficult to find the MAG technology and the welding parameters used by the machine manufacturer for an optimal comparison of the results. However, if we focus on belt damage (Figure 2b), it can be concluded that the violation occurs precisely at the construction boundary. The damage further continues into the heat-affected zone. The same damage processes were observed during tensile tests carried out either in the presented research or in the searched literature. The obtained results may lead to the conclusion that the influence of the microstructure of the welding technology has a fundamental influence on the damage. This postulate resulted in the idea of changing the material of the belt to S960QL steel (which is more profitable for the manufacturer in terms of the quantity of production than the purchase of unconventional welding technology). Therefore, attention was focused on the research of the material parameters of S960QL steel welded both by unconventional laser and electron technologies and by conventional MAG technology. The achieved results in the field of microstructure, hardness and strength [7,10] fully corresponded with the analyzed works [34,35,36], but always with differences at least in the welding parameters or welding technologies themselves. In work [37], the authors observed the presence of residual stresses in the welded joint of S500MC steel, which reached significant values (up to 480 MPa in tension). This value is very close to the yield strength of the base material (see Table 2). The same values were also reached in other works using different conventional welding technologies [38,39]. The research shows that cracks were influenced by the fragile structure in HAZ and the significant concentration of tensile stress in the weld.

Residual stresses are thus another phenomenon fundamentally affecting the desired trouble-free operation of the solved component. In the work [6], the authors focused on monitoring residual stresses after welding S960QL steel with electron and laser beam technologies. Under the defined conditions of the experiment, maximum values of 250 MPa in tension were achieved, which is a significant decrease in comparison with MAG technology for S500MC steel. In future research, we therefore expect to verify the residual stresses of S960QL welded by MAG technology and compare the results of this high-strength steel across conventional and unconventional welding technologies. In the case of significant differences, the residual stresses from MAG are expected to be extremely helpful during the belt breaking process.

The cyclical loading of the belt in operation further resulted in the requirement to experimentally verify the fatigue life of the S500MC and S960QL materials. Currently, if we were to comply with the conservative recommendations of the International Institute of Welding, there would be practically no difference in the fatigue life of the welded constructions S500MC and S960QL, and thus their life would be independent of the yield strength of the base material. Thus, from a practical point of view, the proposed replacement of the belt material would not improve the fatigue life of the structure. That is why it was necessary to examine this situation. From the analysis of the current state of the literature in the field of fatigue of S960QL and its welds by different technologies, significant gaps were observed depending on the input factors. The work most evaluates the fatigue life of the base material S960QL in tension, in addition for low-cycle fatigue [40,41], while the belt of the demining machine is loaded at the point of damage mainly by bending and in a state of tension corresponding to high-cycle fatigue. The value of the maximum loading corresponded to the deterministic driving mode of the machine when reversing up a slope with an angle of 25°. An important insight emerged from the comparison of Figure 2, where the site of belt damage is visible, and Figure 28, where the place with the largest achieved stress distributions (approx. 240 MPa) can be seen. Their location is identical. In the case of the basic material S960QL, this stress corresponds to the permanent service life (over 10^7^ cycles, see Figure 26 and Figure 27), and the same applies to the permanent service life for the basic material S500MC with a determined bending fatigue limit of approximately 235 MPa. For steel S960QL, nothing changes even in the case of using weld joints of conventional (MAG) and non-conventional (electron, laser) welding technologies with permanent fatigue life (for the specified maximum load). The situation is different in the case of welds of S500MC steel. With low numbers of cycles to fracture, the fatigue life is at the limit of the recommended FAT curve. However, it is still true that this stress (approx. 500 MPa) is well above the specified limit of 240 MPa. It is possible to say that the conducted research confirmed the dependence of the fatigue life on the type of steel and also on the welding technology. These results only emphasize the importance of planned research on the quantification of residual stresses. However, the currently achieved results of this study can be directly used in the topological optimization of the belt in order to make the most of the advantages offered by the implementation of high-strength steels in conjunction with experimentally verified service life (for various machine components, both welded and non-welded).

Obtaining the fatigue curves of modern materials requires considerable time. In addition, it was stated in the work [6] that the manufacturer did not identify fatigue damage in the fracture surface of the broken component. This is also supported by the results presented in Section 3.2. Due to economic reasons, the problem must be solved in the shortest possible time; a complex solution to the problem includes a processed structural design ensuring that the maximum load in the belt will be approx. 240 MPa, determined by a combination of analytical and numerical calculations presented in the paper. It is understandable that manufacturers (together with their suppliers) strive to introduce reliable products into prototypes and later series productions. Reducing development costs (and time) will gain comparative advantages from the point of view of the competition.

## 5. Conclusions

In the process of experimental works included in this paper, it was possible to create the following:

- Finalizing the knowledge base of a unique test condition used to measure the fatigue life of materials;
- Methodology of sample testing and numerical models corresponding to real loading conditions;
- Calibration curves of the base materials S960QL (and its electron, laser and MAG welds) and S500MC (and its MAG weld) for bending and torsion loading. The curves presented are used to ensure consistency of results when moving from the laboratory environment to the context of IIW regulations;
- Patented structural design of a new type of clamping mechanism, and its mathematical and numerical model with static FEM analysis;

Based on experimental works, the following was found:

- The maximum stress in the belt link is approximately 240 MPa (in bending) under the considered boundary conditions. This stress corresponds to the continuous fatigue life (more than 10^7^ cycles) for both base materials tested (S960QL, S500MC);
- The electron-welded specimens showed only a minimal and therefore negligible reduction in fatigue life compared to the S960QL base material over the entire range of stress levels;
- Compared to the base material S960QL, the laser-welded specimens showed an approximately 40% reduction in life in the low-cycle fatigue range. The curves became closer as the deformation amplitude decreased. This trend continued up to a strain amplitude of 2.85 × 10^−3^ (corresponding to a stress of 610 MPa), when the curves flattened;
- The MAG-welded specimens showed an approximately 75% reduction in life in the low-cycle fatigue range compared to the base material S960QL. As the deformation amplitude decreased, the curves moved closer together. This trend continued down to the lowest deformation amplitudes measured (S960QL MAG: 4 teeth, 2.88 × 10^−3^, 577 MPa, S960QL base material: 4 teeth, 2.85 × 10^−3^, 610 MPa), when the curves moved most closely together, with a 30% difference;
- More significant differences were also observed when comparing the life of the base material S500MC and its MAG welds. The life curves converged over the full spectrum of load cycles from the maximum deformation amplitudes with a difference of 70% to the minimum measured amplitudes when the difference was approximately 33%. MAG welds of S500MC were found to be close to the recommended FAT curve for low-cycle fatigue (but still considered satisfactory). As the number of cycles to failure increased, this difference became more pronounced and all specimens were well above this curve.

## Figures and Tables

**Table 1:** Chemical composition of S960QL and S500MC materials.

Steel	Chemical Composition—Max. wt. [%]							
S960QL	C	Si	Mn	B	Nb	Cr	V	Cu
	0.16	0.21	1.24	0.001	0.015	0.2	-	0.01
	Ti	Al	Mo	Ni	N	P	S	
	0.004	0.06	0.602	0.06	0.003	0.01	0.001	
S500MC	C	Si	Mn	B	Nb	Cr	V	Cu
	0.17	0.28	1.24	-	0.018	-	0.004	-
	Ti	Al	Mo	Ni	N	P	S	
	0.02	0.026	-	-	-	0.019	0.017	

**Table 4:** The eccentric deflection and the corresponding angle of rotation twist angles of the test sample are fixed by the construction for the eccentricity of 4 mm.

Eccentric Tooth (exc.4 mm) [-]	Deviation [mm]	Twist Angle [rad]	Eccentric Tooth (exc.4 mm) [-]	Deviation [mm]	Twist Angle [rad]
1	0.6784	0.0048	10	6.0054	0.0429
2	1.3520	0.0097	11	6.4320	0.0459
3	2.0158	0.0144	12	6.8122	0.0486
4	2.6651	0.0190	13	7.1434	0.0510
5	3.2952	0.0235	14	7.4231	0.0530
6	3.9016	0.0279	15	7.6493	0.0546
7	4.4798	0.0320	16	7.8204	0.0558
8	5.0258	0.0359	17	7.9352	0.0566
9	5.5355	0.0395	18	7.9928	0.0570

**Table 5:** The eccentric deflection and the corresponding twist angles of the test sample are fixed by the construction for the eccentricity of 2 mm.

Eccentric Tooth (exc.2 mm) [-]	Deviation [mm]	Twist Angle [rad]	Eccentric Tooth (exc.2 mm) [-]	Deviation [mm]	Twist Angle [rad]
1	0.3392	0.0022	10	3.0027	0.0200
2	0.6760	0.0045	11	3.2160	0.0214
3	1.0079	0.0067	12	3.4061	0.0227
4	1.3326	0.0089	13	3.5717	0.0238
5	1.6476	0.0110	14	3.7116	0.0247
6	1.9508	0.0130	15	3.8247	0.0255
7	2.2399	0.0149	16	3.9102	0.0261
8	2.5129	0.0168	17	3.9676	0.0264
9	2.7678	0.0184	18	3.9964	0.0266

**Table 6:** Input data for creating the corresponding material model.

E [GPa]	μ [-]	Re [MPa]	E_T_ [MPa]
210	0.3	1049 for S960QL 521 for S500MC	5000

**Table 7:** Numerical values from analysis of torsional loading with eccentricity e = 4 mm for S960QL material and its electron and laser beam welds.

Tooth [-]	γ_xy_ [10^−3^]	τ_xy_ [MPa]	Tooth [-]	γ_xy_ [10^−3^]	τ_xy_ [MPa]
1	0.83	67.27	10	7.37	594.00
2	1.66	134.07	11	7.91	606.84
3	2.47	199.90	12	8.56	610.12
4	3.27	264.31	13	9.24	613.44
5	4.05	326.81	14	9.91	616.72
6	4.79	386.99	15	10.54	619.74
7	5.50	444.34	16	11.06	622.23
8	6.17	498.52	17	11.43	623.99
9	6.80	549.12	18	11.63	624.92

**Table 8:** Numerical values from analysis of bending loading with eccentricity e = 2 mm for S960QL material and its electron and laser beam welds.

Tooth [-]	ε_xx_ [10^−3^]	σ_xx_ [MPa]	Tooth [-]	ε_xx_ [-]	σ_xx_ [MPa]
1	0.72	153.69	10	6.86	1130.16
2	1.44	307.45	11	7.55	1144.64
3	2.15	459.96	12	8.19	1157.47
4	2.85	610.24	13	8.78	1168.84
5	3.53	756.97	14	9.29	1178.43
6	4.19	899.14	15	9.71	1186.35
7	4.82	1035.43	16	10.04	1192.18
8	5.45	1096.69	17	10.25	1196.17
9	6.17	1114.70	18	10.36	1198.07

**Table 9:** Numerical values from analysis of torsional loading with eccentricity e = 4 mm for arc-welded S960QL material.

Tooth [-]	γ_xy_ [10^−3^]	τ_xy_ [MPa]	Tooth [-]	γ_xy_ [10^−3^]	τ_xy_ [MPa]
1	0.88	48.30	10	7.39	573.44
2	1.76	102.52	11	7.93	584.75
3	2.57	160.26	12	8.57	586.14
4	3.37	231.74	13	9.26	589.80
5	4.12	292.06	14	9.92	592.83
6	4.90	359.43	15	10.56	596.81
7	5.59	420.59	16	11.07	599.18
8	6.21	482.78	17	11.41	601.97
9	6.82	532.66	18	11.65	602.88

**Table 10:** Numerical values from analysis of bending loading with eccentricity e = 2 mm for arc-welded S960QL material.

Tooth [-]	ε_xx_ [10^−3^]	σ_xx_ [MPa]	Tooth [-]	ε_xx_ [-]	σ_xx_ [MPa]
1	0.77	126.85	10	6.90	1114.44
2	1.48	272.08	11	7.59	1121.96
3	2.19	423.14	12	8.23	1132.14
4	2.88	577.87	13	8.80	1147.38
5	3.57	713.19	14	9.31	1156.08
6	4.22	855.76	15	9.73	1165.27
7	4.85	978.55	16	10.05	1182.30
8	5.48	1050.62	17	10.27	1185.17
9	6.20	1082.27	18	10.37	1187.39

**Table 11:** Numerical values from analysis of torsional loading with eccentricity e = 4 mm for the base material S500MC.

Tooth [-]	γ_xy_ [10^−3^]	τ_xy_ [MPa]	Tooth [-]	γ_xy_ [10^−3^]	τ_xy_ [MPa]
1	0.86	32.74	10	7.39	307.54
2	1.73	63.86	11	7.91	327.48
3	2.55	97.14	12	8.57	350.17
4	3.31	128.47	13	9.25	364.23
5	4.09	166.13	14	9.92	368.87
6	4.84	197.48	15	10.55	369.16
7	5.55	233.79	16	11.07	369.92
8	6.20	258.93	17	11.42	370.28
9	6.82	282.36	18	11.64	370.98

**Table 12:** Numerical values from analysis of bending loading with eccentricity e = 2 mm for base material S500MC.

Tooth [-]	ε_xx_ [10^−3^]	σ_xx_ [MPa]	Tooth [-]	ε_xx_ [-]	σ_xx_ [MPa]
1	0.79	73.32	10	6.95	580.13
2	1.50	152.76	11	7.63	587.62
3	2.21	232.19	12	8.27	591.31
4	2.94	308.96	13	8.83	602.11
5	3.60	381.14	14	9.33	609.03
6	4.26	467.43	15	9.76	614.75
7	4.88	526.20	16	10.08	620.44
8	5.53	561.22	17	10.29	626.09
9	6.24	576.82	18	10.39	628.40

**Table 13:** Numerical values from analysis of torsional loading with eccentricity e = 4 mm for the arc-welded material S500MC.

Tooth [-]	γ_xy_ [10^−3^]	τ_xy_ [MPa]	Tooth [-]	γ_xy_ [10^−3^]	τ_xy_ [MPa]
1	0.88	30.58	10	7.40	256.40
2	1.76	56.14	11	7.93	268.15
3	2.59	84.59	12	8.59	288.61
4	3.35	114.22	13	9.26	300.53
5	4.14	153.93	14	9.94	310.09
6	4.88	176.84	15	10.57	318.52
7	5.58	190.04	16	11.08	323.18
8	6.23	211.52	17	11.43	323.99
9	6.86	229.96	18	11.64	324.41

**Table 14:** Numerical values from analysis of bending loading with eccentricity e = 2 mm for arc-welded S500MC material.

Tooth [-]	ε_xx_ [10^−3^]	σ_xx_ [MPa]	Tooth [-]	ε_xx_ [-]	σ_xx_ [MPa]
1	0.84	55.83	10	6.00	498.47
2	1.53	117.44	11	7.69	512.81
3	2.24	172.54	12	8.32	519.42
4	2.99	235.05	13	8.88	524.07
5	3.63	284.63	14	9.39	529.40
6	4.29	341.88	15	9.80	533.22
7	4.92	390.14	16	10.11	535.05
8	5.58	442.62	17	10.32	536.70
9	6.27	473.29	18	10.44	537.00

**Table 15:** Technical parameters of the pumps used in the demining machine Božena 5.

	Symbol	Value	Unit
Maximum pump volume	V_Gmax_	125	cm^3^
Maximum flow rate	q_vmax_	356	l/min
Work pressure	p	400	bar
Efficiency	η_p_	0.93	-

**Table 16:** Technical parameters of the hydraulic motors used in the demining machine Božena 5.

	Symbol	Value	Unit
Maximum pump volume	V_GMmax_	63	cm^3^
Maximum flow rate	q_vm max_	315	L/min
Work pressure	p	400	bar
Efficiency	η_m_	0.925	-

**Table 17:** Material parameters of numerical calculation.

E [GPa]	μ [-]	Re [MPa]	ρ [kg·m^−3^]
210	0.33	500	7850

**Table 18:** Input parameters of the functional calculation.

Parameter	Parameter Unit	Parameter Value	Parameter Meaning
η_p_	[-]	0.8	mechanical efficiency of the wheel gearbox
i_p_	[-]	38.6	gear ratio of the wheel gearbox
r	[m]	0.465	pitch radius of the chain wheel (rosettes)
m	[kg]	10,240	the weight of the Bozen 5
m_f_	[kg]	2850	the weight of the periphery of the ground cutter
α	[°]	25	maximum climb of the vehicle
α_1_	[°]	32	the angle of deviation of the normal force from the driving force
m_b_	[kg]	12.2	weight of one belt link
n	[ot·s^−1^]	0.66	revolutions of the rosette at the maximum speed of the vehicle
f	[-]	0.1	friction coefficient of the kinematic pair belt–rosette

**Table 2:** Determined average values of the mechanical properties of the tested materials and their welds.

Sample Material	YS [MPa]	UTS [MPa]	A [%]
Base material S960QL	1035.45	1077.75	11.625
Base material S500MC	521	630.7	20.5
S960QL laser-welded	1053.5	1093.3	11.15
S960QL electron-welded	1057.8	1097.3	11.16
S960QL arc-welded	823.5	930.8	4.3
S500MC arc-welded	374.5	523.4	6.9

**Table 3:** Welding parameters of the technologies used.

**MAG—S960QL**					
		**Welding Current I [A]**	**Welding Voltage U [V]**	**Welding Speed v [mm/s]** **2.5**	**Heat Input Q [kJ/mm]**
Root pass		164	23.3	2.5	1.67
Cap pass		230	28.2	3.1	
**MAG—S500MC**					
		**Welding current I [A]**	**Welding voltage U [V]**	**Welding speed v [mm/s]** **2.5**	**Heat input Q [kJ/mm]**
Root pass		150	25	2.9	1.3
Cap pass		168	26	2.7	
**Laser welding**					
**Power P [W]**		**Speed** **v [mm/s]**	**Focus location [mm]**	**Thread diameter [mm]**	**Heat input Q [kJ/mm]**
5000		7.5	−4	0.1	0.63
**Electron welding**					
**Welding current I [mA]**	**Acceleration voltage U [kV]**	**Speed** **v [mm/s]**	**Focus location [mm]**	**Vacuum ratio [Pa]**	**Heat input Q [kJ/mm]**
115	55	11	0	0.09	0.55

**Table 19:** Average number of cycles to fracture of base material S960QL loaded by torsion.

The Tooth of the Eccentric Pair [-]	Number of Measurements at the Given Level [-]	Average Number of Cycles N_f_ [-] to Fracture	The Tooth of the Eccentric Pair [-]	Number of Measurements at the Given Level [-]	Average Number of Cycles N_f_ [-] to Fracture
11	3	18,820	7	6	383,958
10	3	26,125	6	5	2,709,740
9	4	39,129	5	4	13,949,911
8	3	147,690	-	-	-

**Table 20:** Average number of cycles to fracture of base material S500MC loaded by torsion.

The Tooth of the Eccentric Pair [-]	Number of Measurements at the Given Level [-]	Average Number of Cycles N_f_ [-] to Fracture	The Tooth of the Eccentric Pair [-]	Number of Measurements at the Given Level [-]	Average Number of Cycles N_f_ [-] to Fracture
10	3	2163	5	4	167,364
8	3	4955	4	4	2,311,323
6	3	6223	3	3	7,058,667

**Table 21:** Average cycles to fracture of S960QL laser-welded and loaded by torsion.

The Tooth of the Eccentric Pair [-]	Number of Measurements at the Given Level [-]	Average Number of Cycles N_f_ [-] to Fracture	The Tooth of the Eccentric Pair [-]	Number of Measurements at the Given Level [-]	Average Number of Cycles N_f_ [-] to Fracture
8	4	19,796	5	4	374,498
7	6	28,353	4	4	12,775,224
6	5	129,650	-	-	-

**Table 22:** Average number of cycles to fracture of S960QL material welded by electron and loaded by torsion.

The Tooth of the Eccentric Pair [-]	Number of Measurements at the Given Level [-]	Average Number of Cycles N_f_ [-] to Fracture	The Tooth of the Eccentric Pair [-]	Number of Measurements at the Given Level [-]	Average Number of Cycles N_f_ [-] to Fracture
9	5	19,853	6	5	1,065,550
8	4	48,588	5	3	11,198,761
7	6	460,378	-	-	-

**Table 23:** Average cycles to fracture of S960QL MAG-welded and loaded by torsion.

The Tooth of the Eccentric Pair [-]	Number of Measurements at the Given Level [-]	Average Number of Cycles N_f_ [-] to Fracture	The Tooth of the Eccentric Pair [-]	Number of Measurements at the Given Level [-]	Average Number of Cycles N_f_ [-] to Fracture
8	3	13,121	5	4	350,113
7	5	22,221	4	3	10,552,429
6	5	108,654	-	-	-

**Table 24:** Average cycles to fracture of MAG-welded S500MC and loaded by torsion.

The Tooth of the Eccentric Pair [-]	Number of Measurements at the Given Level [-]	Average Number of Cycles N_f_ [-] to Fracture	The Tooth of the Eccentric Pair [-]	Number of Measurements at the Given Level [-]	Average Number of Cycles N_f_ [-] to Fracture
10	3	1406	5	4	127,197
8	4	3419	4	5	1,849,058
6	3	4543	3	4	5,435,174

**Table 25:** Average number of cycles to fracture of MAG-welded material S960QL loaded by bending.

The Tooth of the Eccentric Pair [-]	Number of Measurements at the Given Level [-]	Average Number of Cycles N_f_ [-] to Fracture	The Tooth of the Eccentric Pair [-]	Number of Measurements at the Given Level [-]	Average Number of Cycles N_f_ [-] to Fracture
11	3	2844	6	5	151,892
9	3	4912	5	5	3,613,909
8	3	6138	4	3	8,858,825
7	4	15,401	-	-	-

**Table 26:** Average number of cycles to fracture of base material S960QL loaded by bending.

The Tooth of the Eccentric Pair [-]	Number of Measurements at the Given Level [-]	Average Number of Cycles N_f_ [-] to Fracture	The Tooth of the Eccentric Pair [-]	Number of Measurements at the Given Level [-]	Average Number of Cycles N_f_ [-] to Fracture
11	3	13,941	6	6	288,086
9	3	19,902	5	4	6,943,261
8	4	24,517	4	3	13,017,934
7	3	40,881	-	-	-

**Table 27:** Average number of cycles to fracture of base material S500MC loaded by bending.

The Tooth of the Eccentric Pair [-]	Number of Measurements at the Given Level [-]	Average Number of Cycles N_f_ [-] to Fracture	The Tooth of the Eccentric Pair [-]	Number of Measurements at the Given Level [-]	Average Number of Cycles N_f_ [-] to Fracture
10	3	1321	5	4	2,113,422
8	3	2242	4	3	7,328,114
7	3	13,150	3	3	14,874,662
6	4	62,478	-	-	-

**Table 28:** Average cycles to fracture of S960QL laser-welded and loaded by bending.

The Tooth of the Eccentric Pair [-]	Number of Measurements at the Given Level [-]	Average Number of Cycles N_f_ [-] to Fracture	The Tooth of the Eccentric Pair [-]	Number of Measurements at the Given Level [-]	Average Number of Cycles N_f_ [-] to Fracture
11	3	9667	6	5	523,216
9	3	11,653	5	3	1,928,540
8	3	12,952	4	3	13,458,760
7	4	40,181	-	-	-

**Table 29:** Average number of cycles to fracture of S960QL material welded by electron and loaded by bending.

The Tooth of the Eccentric Pair [-]	Number of Measurements at the Given Level [-]	Average Number of Cycles N_f_ [-] to Fracture	The Tooth of the Eccentric Pair [-]	Number of Measurements at the Given Level [-]	Average Number of Cycles N_f_ [-] to Fracture
11	4	12,413	6	6	326,631
9	3	17,023	5	4	6,524,119
8	4	18,477	4	3	12,143,814
7	5	29,710	-	-	-

**Table 30:** Average cycles to fracture of S960QL material MAG-welded and loaded by bending.

The Tooth of the Eccentric Pair [-]	Number of Measurements at the Given Level [-]	Average Number of Cycles N_f_ [-] to Fracture	The Tooth of the Eccentric Pair [-]	Number of Measurements at the Given Level [-]	Average Number of Cycles N_f_ [-] to Fracture
11	3	2844	6	5	151,892
9	3	4912	5	5	3,613,909
8	3	6138	4	3	8,858,825
7	4	15,401	-	-	-

**Table 31:** Average cycles to fracture of S960QL material MAG-welded and loaded by torsion.

The Tooth of the Eccentric Pair [-]	Number of Measurements at the Given Level [-]	Average Number of Cycles N_f_ [-] to Fracture	The Tooth of the Eccentric Pair [-]	Number of Measurements at the Given Level [-]	Average Number of Cycles N_f_ [-] to Fracture
8	3	695	5	4	888,979
7	5	4734	4	3	4,396,868
6	5	32,622	3	3	10,114,768

**Table 32:** Mathematical models of squared form of Wöhler and Manson–Coffin curves of test materials and their welds stressed by cyclic bending.

**S960QL—Cyclic Bending**			
	$\sigma_{a}$	$\varepsilon_{a}$	**R^2^**
Base material	$\sigma_{a}=2454.1\cdot {N_{f}}^{-0.08}$	$\varepsilon_{a}=16.696\cdot 10^{-3}\cdot {N_{f}}^{-0.106}$	0.8502
Electron weld	$\sigma_{a}=2337.4\cdot {N_{f}}^{-0.077}$	$\varepsilon_{a}=15.524\cdot 10^{-3}\cdot {N_{f}}^{-0.101}$	0.8356
Laser weld	$\sigma_{a}=2439.8\cdot {N_{f}}^{-0.083}$	$\varepsilon_{a}=16.743\cdot 10^{-3}\cdot {N_{f}}^{-0.11}$	0.8736
Arc weld	$\sigma_{a}=1681.5\cdot {N_{f}}^{-0.07}$	$\varepsilon_{a}=14.884\cdot N_{f}^{-0.105}$	0.8401
**S500MC—Cyclic Bending**			
	$\sigma_{a}$	$\varepsilon_{a}$	**R^2^**
Base material	$\sigma_{a}=1292.7\cdot {N_{f}}^{-0.095}$	$\varepsilon_{a}=1111.9\cdot N_{f}^{-0.083}$	0.8973
Arc weld	$\sigma_{a}=773.41\cdot {N_{f}}^{-0.083}$	$\varepsilon_{a}=9.9984\cdot N_{f}^{-0.086}$	0.8929

**Table 33:** Mathematical models of squared form of Wöhler and Manson–Coffin curves of test materials and their welds stressed by cyclic torsion.

**S960QL—Cyclic Torsion**			
	$\tau_{a}$	$\gamma_{a}$	**R^2^**
Base material	$\tau_{a}=1363.6\cdot {N_{f}}^{-0.086}$	$\gamma_{a}=17.694\cdot 10^{-3}\cdot {N_{f}}^{-0.09}$	0.8913
Electron weld	$\tau_{a}=1182.7\cdot {N_{f}}^{-0.079}$	$\gamma_{a}=14.64\cdot 10^{-3}\cdot {N_{f}}^{-0.079}$	0.9470
Laser weld	$\tau_{a}=1133.3\cdot {N_{f}}^{-0.091}$	$\gamma_{a}=14.029\cdot 10^{-3}\cdot {N_{f}}^{-0.091}$	0.8883
Arc weld	$\tau_{a}=994.03\cdot {N_{f}}^{-0.094}$	$\gamma_{a}=16.511\cdot N_{f}^{-0.082}$	0.8825
**S500MC—Cyclic Torsion**			
	$\sigma_{a}$	$\varepsilon_{a}$	**R^2^**
Base material	$\tau_{a}=669.83\cdot {N_{f}}^{-0.118}$	$\gamma_{a}=15.536\cdot N_{f}^{-0.113}$	0.9203
Arc weld	$\tau_{a}=566.64\cdot {N_{f}}^{-0.123}$	$\gamma_{a}=14.675\cdot N_{f}^{-0.111}$	0.9017

**Table 34:** Calculation results of the compiled analytical model.

Equation No.	Designation of the Calculated Quantity	The Meaning of the Calculated Quantity	Unit	Value
(4)	q_v_	volume flow of hydraulic fluid through the hydraulic pump	[L·min^−1^]	209.25
(5)	q_vm_	volumetric flow rate of hydraulic fluid per hydraulic motor	[L·min^−1^]	104.625
(6)	n	revolutions of the hydraulic motor	[ot·min^−1^]	1536.16
(7)	P_k_	power corresponding to one hydraulic motor	[kW]	54.61
(8)	M_k_	hydraulic motor torque	[N·m]	339.5
(9)	M_kv_	torque at the output of the wheel gearbox	[N·m]	10,483.76
(10)	F_p_	the resulting force acting on the belt is transmitted by a rosette located on the output shaft of the gearbox	[N]	22,545.72
(11)	F_xp_	reduction in force Fp to the hub of the wheel in the direction of travel of the machine	[N]	11,272.86
(12)	F_x max_	the weight of vehicles tilted in the direction of travel of the vehicle (driving mode reversing on a slope)	[N]	54,269.64
(13)	F_x_	the resultant longitudinal force acting on the wheel in its hub	[N]	38,407.68
(14)	F_v_	tangential force in the contact of the rosette with the belt	[N]	1991.24
(15)	F_o_	centrifugal force acting on the belt pin when engaged with a rotating rosette	[N]	97.56
(16)	F_t_	frictional force between belt and rosette	[N]	318.66
(17)	F_n_	normal force of belt–rosette contact	[N]	3186.6
(18)	F_N_	belt tensioning force	[N]	1770.14
(19)	F_vn_	resulting belt tension force	[N]	49,028.52

## Data Availability

The raw data supporting the conclusions of this article will be made available by the authors on request.
